# Computational and Data‐Driven Strategies for Lignocellulosic Biomass Valorization: From Multiscale Modeling to Digitalized Biorefinery Design

**DOI:** 10.1002/advs.78026

**Published:** 2026-09-27

**Authors:** Abdullahi Bello Umar, Jiaxian Zheng, Xiangfeng Lin, Hongwu Liao, Haoqian Xu, Yufei Huang, Zaharaddeen Nasiru Garba, Adamu Uzairu, Weiwei Zhao, Zhanhui Yuan

**Affiliations:** ^1^ College of Materials Engineering Fujian Agriculture and Forestry University Fuzhou People's Republic of China; ^2^ Department of Chemistry Faculty of Physical Sciences Ahmadu Bello University Zaria Kaduna Nigeria; ^3^ Birmingham Centre for Energy Storage (BCES) & School of Chemical Engineering University of Birmingham Edgbaston UK

**Keywords:** biomass valorization, density functional theory, digital biorefinery, lignocellulosic biomass, machine learning, molecular dynamics, multiscale modeling

## Abstract

Lignocellulosic biomass is a major renewable carbon resource for sustainable production of fuels, chemicals, and functional materials. Yet its valorization remains constrained by structural recalcitrance, feedstock heterogeneity, and the coupling of molecular and process phenomena. This review critically examines computational and data‐driven strategies for predictive biorefinery development, with emphasis on how experimentally anchored information can be transferred across scales. Structural determinants of deconstructability, including cellulose organization, lignin chemistry, lignin‐carbohydrate interactions, porosity, and accessibility, are assessed together with density functional theory, molecular dynamics, reactive simulations, thermodynamic and kinetic modeling, and machine learning. Particular attention is given to computational solvent design, catalytic upgrading, pyrolysis reaction networks, reactor‐scale coupling, and the limitations imposed by model simplification, dataset heterogeneity, extrapolation, and uncertainty. Representative multiscale case studies illustrate validated information handoffs from detailed kinetics to CFD, molecular screening to experiment, and pretreatment to life‐cycle assessment. FAIR data infrastructure, workflow automation, and digital twins are discussed as enabling layers. Future progress depends on experimentally validated scale transfer, multicycle durability of solvents and catalysts, uncertainty‐aware modeling, and interoperable data ecosystems for practical biorefinery design.

## Graphical abstract

This Review critically connects structural recalcitrance, molecular modeling, solvent and catalyst design, pyrolysis, machine learning, reactor simulation, and FAIR digital infrastructure. Emphasis is placed on experimentally validated information transfer across scales, uncertainty and applicability domains, and the recycle, durability, techno‐economic, and life‐cycle constraints required to move computational insights toward predictive lignocellulosic biorefinery design.

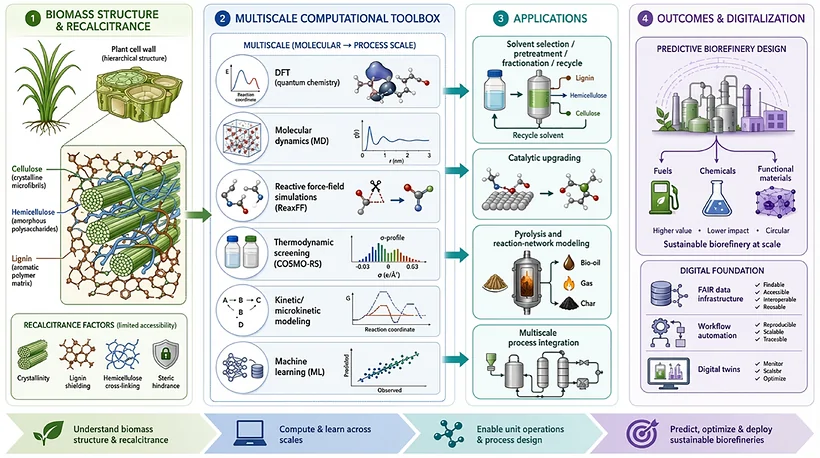

## 1 Introduction

Lignocellulosic biomass is increasingly recognized as one of the most strategically important renewable carbon resources for the transition from a fossil‐based economy to a circular, low‐emission chemical industry. Its abundance, broad geographical distribution, and lack of competition with food supply chains make it an attractive feedstock for producing fuels, platform chemicals, polymers, and advanced functional materials in integrated biorefineries [1, 2, 3, 4, 5, 6]. Yet efficient lignocellulose valorization remains far from straightforward. The central challenge is not the lack of viable reaction pathways, but the difficulty of controlling biomass deconstruction and upgrading in a selective, energy‐efficient, and economically viable manner across multiple interconnected scales [4, 7, 8, 9, 10]. Consequently, the field has evolved from a conversion‐centered discipline into a system‐level problem in which pretreatment, catalytic upgrading, separations, solvent recovery, techno‐economic assessment, and environmental performance must be evaluated as interdependent components of an integrated process [5, 11, 12, 13]

The origin of this challenge lies in the structural organization of plant cell walls. Lignocellulosic biomass is not a simple mixture of cellulose, hemicellulose, and lignin, but a hierarchically assembled composite in which these constituents are coupled through hydrogen bonding, hydrophobic interactions, covalent cross‐links, and spatial confinement [14, 15, 16]. Cellulose forms semicrystalline microfibrils stabilized by dense intra‐ and intermolecular hydrogen‐bonding networks that restrict solvent penetration and reduce access to glycosidic bonds [17, 18, 19, 20]. Hemicellulose introduces further heterogeneity through its branched, amorphous, and feedstock‐dependent character. In contrast, lignin forms a chemically resilient aromatic matrix that modifies wetting behavior, shields polysaccharides, and participates in lignin‐carbohydrate interactions that reinforce recalcitrance [21, 22, 23]. These barriers do not act independently. Rather, recalcitrance emerges from the coupling of molecular interactions with nanoscale interfaces, mesoscale packing, pore accessibility, and macroscopic transport constraints. Recent structural studies support this view by showing that lignin can self‐associate into hydrophobic nanodomains, that xylan‐cellulose association depends strongly on substitution pattern and conformation, and that lignin‐carbohydrate interactions evolve during wall maturation [24, 25, 26].

This multiscale complexity has made computational and data‐driven methods increasingly central to biomass research. Over the past decade, density functional theory has provided mechanistic insight into bond activation, adsorption, catalytic hydrogenolysis, hydrodeoxygenation, and depolymerization of lignin model compounds [27, 28, 29, 30, 31, 32]. Molecular dynamics simulations have clarified solvation behavior, hydrogen bond disruption, cellulose and lignin organization, lignin aggregation, biomass‐solvent interactions, and solvent‐assisted deconstruction [9, 20, 33, 34, 35, 36, 37, 38]. Reactive simulation approaches, particularly ReaxFF‐based molecular dynamics, have extended atomistic analysis to larger and chemically evolving systems, particularly in pyrolysis and thermochemical conversion, where predefined pathways often fail to capture radical chemistry, bond rearrangement, and secondary transformations [39, 40, 41, 42, 43, 44, 45, 46]. In parallel, thermodynamic screening and computational solvent‐design strategies have expanded the exploration of ionic liquids, deep eutectic solvents, organosolv systems, and other fractionation media [33, 36, 47, 48, 49, 50, 51]. Machine learning has also emerged as a powerful accelerator for catalyst discovery, solvent selection, property prediction, kinetic modeling, and process optimization [39, 52, 53, 54, 55, 56]. These developments have shifted the literature from isolated mechanistic case studies toward integrated workflows that combine quantum chemistry, molecular simulation, thermodynamic screening, kinetic modeling, and statistical learning to navigate the highly coupled design space of biomass conversion systems [10, 39, 53, 54, 57].

Despite this progress, the central translational challenge remains unresolved. The literature is still fragmented across methods, model systems, and unit operations. High‐level mechanistic studies often focus on idealized substrates, simplified solvent environments, or selected catalyst surfaces, yielding valuable but localized insight [27, 29, 32]. By contrast, process‐level assessments frequently rely on empirical, lumped, or black‐box descriptions that obscure the molecular origins of experimentally observed limitations [58, 59, 60]. In this Review, the central translational challenge is defined more specifically as the difficulty of propagating mechanistic predictions obtained from idealized molecular models or laboratory conditions into quantitative performance predictions for chemically heterogeneous feedstocks under process‐relevant conditions while preserving model validity and uncertainty. The relevant question is therefore not only whether a solvent dissolves lignin, a catalyst cleaves a β‐O‐4 linkage, or an ML model fits a dataset, but whether the resulting advantage survives changes in feedstock, solvent recycle, catalyst lifetime, transport, separation, and scale [11, 13, 47, 48, 61, 62].

**FIGURE 1:**
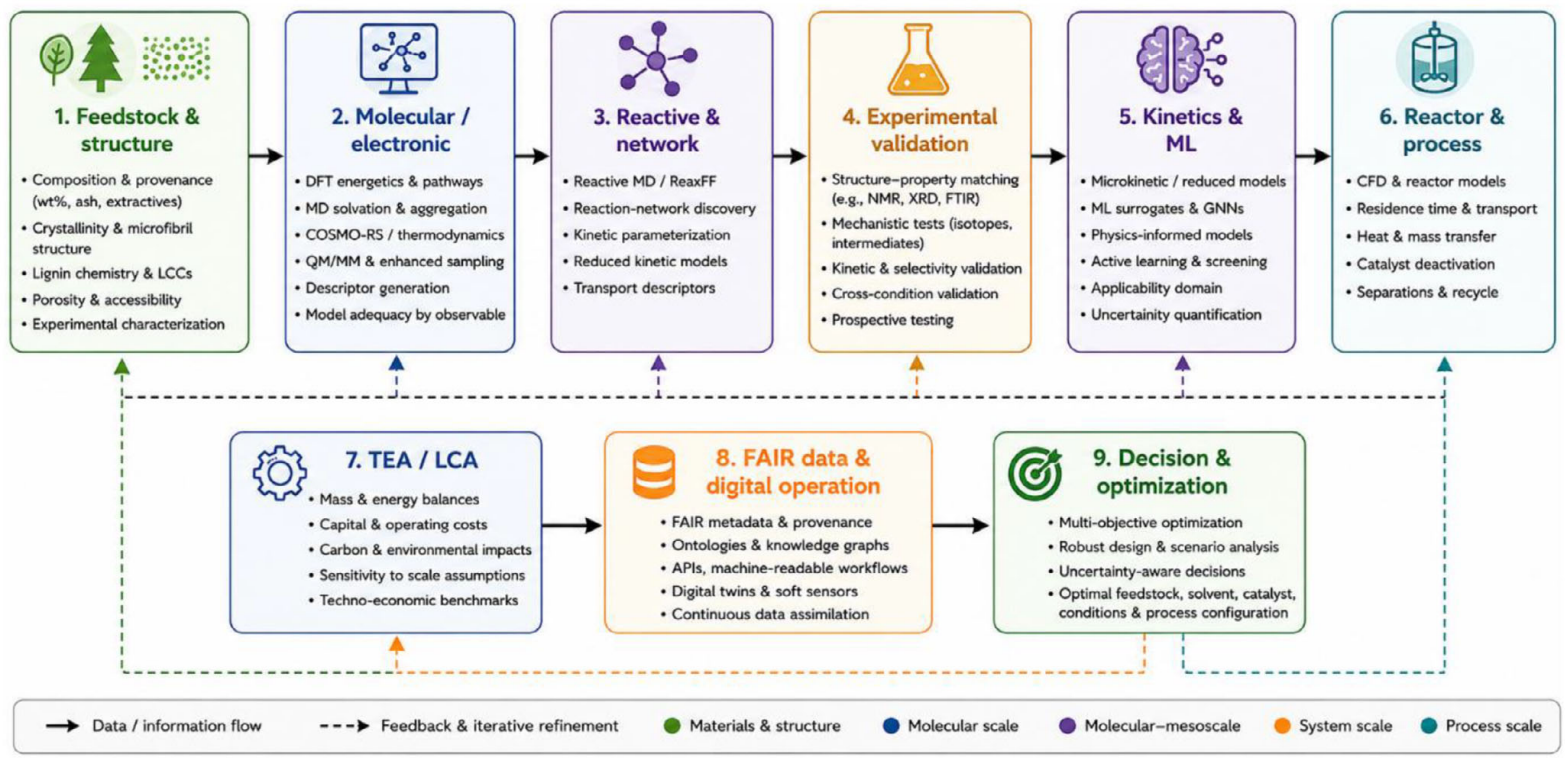
Overall predictive multiscale workflow for computational and data‐driven lignocellulosic biomass valorization. Information flows from feedstock characterization and molecular/electronic modeling through reactive and network modeling, experimental validation, kinetic and ML models, reactor and process simulation, TEA/LCA, and FAIR‐data‐enabled digital operation. Experimental feedback, model refinement, process constraints, uncertainty propagation, provenance, and domain‐of‐applicability assessment operate across the complete workflow.

Accordingly, this review adopts a multiscale and data‐driven perspective on lignocellulosic biomass valorization, with particular emphasis on how computational representations and predictive frameworks can be connected across levels of description. First, we examine the structural origins of biomass recalcitrance and the representations used to describe them [15, 16, 26, 63]. We then assess the capabilities and limitations of density functional theory, molecular dynamics, reactive force fields, thermodynamic screening, kinetic modeling, and process simulation in relation to solvent selection, pretreatment, catalytic upgrading, and thermochemical conversion [28, 33, 53, 58, 64]. Machine learning is considered as an integrative layer for screening, surrogate modeling, descriptor discovery, and uncertainty‐aware prediction when grounded in chemically meaningful data [54, 55, 65, 66]. Finally, FAIR data infrastructure, ontologies, knowledge graphs, open reaction databases, workflow automation, and digital‐twin concepts are examined as enabling components of reproducible and scalable biorefineries [67, 68, 69]. Figure 1 summarizes the resulting predictive workflow. In this framework, scale bridging refers to the transfer of a physically defined quantity or validated relationship between levels of description, while decision relevance requires that the transferred information can alter an experimental, reactor, process, economic, or environmental choice.

## 2 Structural Complexity and Recalcitrance of Lignocellulosic Biomass

**FIGURE 2:**
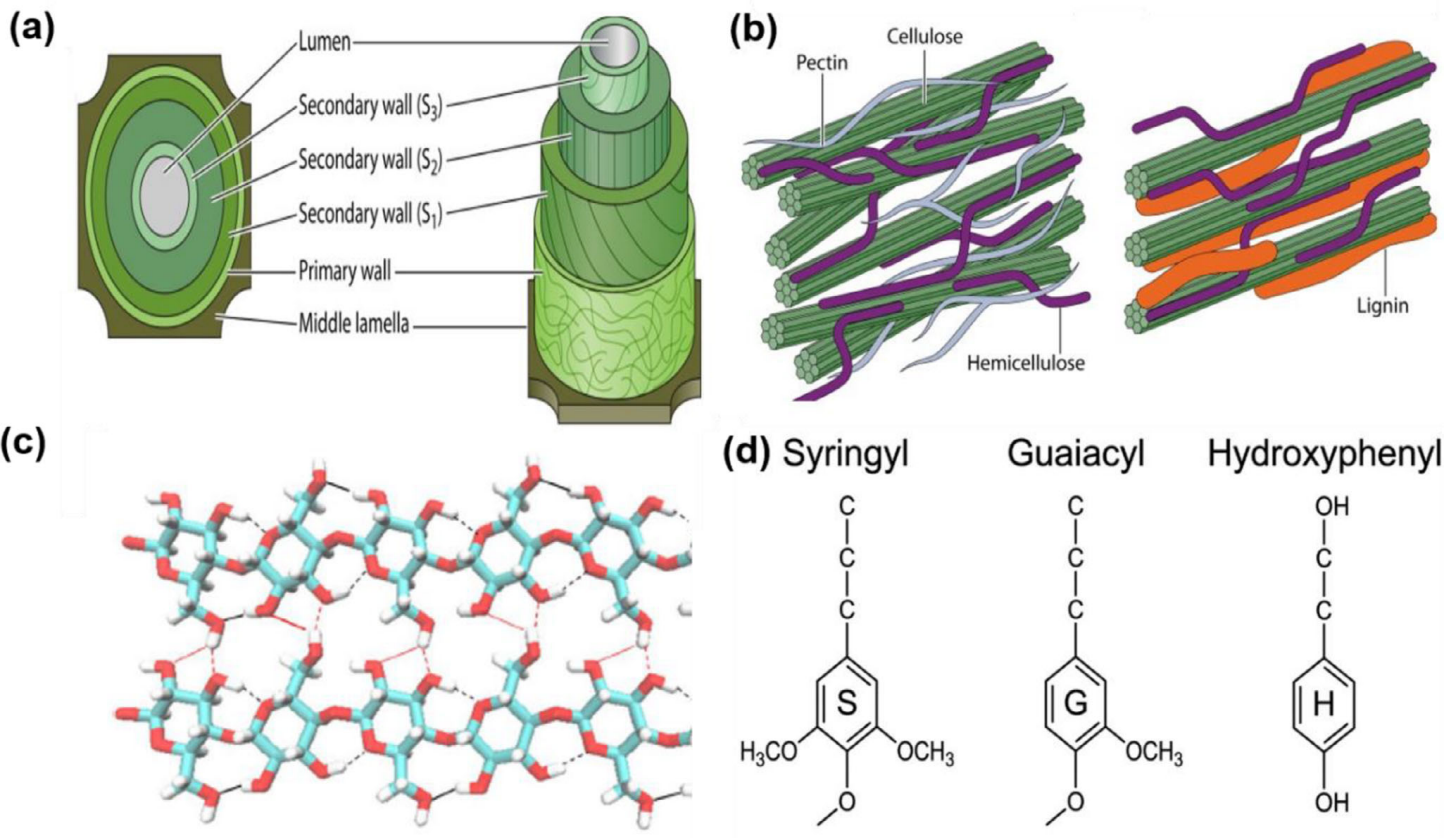
(a) Plant tissue heterogeneity and cell‐wall stratification; (b) cellulose microfibrils embedded in a hemicellulose‐lignin matrix. Panels (a,b) reproduced from Ref. [70] with permission from the American Society for Microbiology. Copyright 2014, American Society for Microbiology. (c) Dense hydrogen‐bonding networks governing cellulose crystallinity. Reproduced from Ref. [71] under the terms of the Creative Commons Attribution 3.0 Unported Licence (CC BY 3.0). 2018 The Authors. Published by the Royal Society of Chemistry. (d) Lignin monomeric diversity (H, G, and S units) forming a cross‐linked polyphenolic framework.

Lignocellulosic biomass derives its resistance to chemical, thermal, and biological deconstruction from a hierarchical cell‐wall architecture that has evolved to provide mechanical strength, controlled hydration, and long‐term durability. As illustrated in Figure 2, the plant cell wall is not a simple mixture of cellulose, hemicellulose, and lignin, but a spatially organized composite extending from polymer‐chain packing and microfibril assembly at the nanometer scale to lamellar, wall, and tissue‐level organization at larger scales [1, 10, 16]. Recalcitrance should therefore be understood not merely as low intrinsic reactivity, but as a systems‐level property that increases energy demand, restricts fractionation efficiency, complicates separations, and narrows the operating window for downstream upgrading. Recent structural studies have substantially revised the classical picture of the wall by showing that lignin can self‐associate into hydrophobic nanodomains, that xylan‐cellulose contacts depend strongly on xylan conformation and substitution pattern, and that lignin‐carbohydrate mixing evolves during wall maturation rather than remaining compositionally static [10, 24, 26].

### 2.1 Composition and Hierarchical Organization

Cellulose, hemicellulose, and lignin remain the three principal structural constituents of lignocellulose. Still, their importance lies less in their isolated identities than in how they are spatially arranged and mutually constrained within the wall. At the tissue level, biomass heterogeneity arises from differences in cell type, lumen size, wall thickness, and the relative proportions of primary and secondary wall material, all of which influence deconstructability (Figure 2a). At the wall level, cellulose microfibrils are embedded within a surrounding matrix of hemicellulose, pectin, and lignin, generating a tightly integrated composite rather than a simple polymer blend (Figure 2b). Cellulose consists of linear β‐(1→4)glucan chains that aggregate into elementary fibrils and then microfibrils stabilized by dense intra‐ and intermolecular hydrogen bonding. These microfibrils provide the main load‐bearing framework and contribute strongly to tensile strength, reduced swelling, and limited solvent accessibility [10, 21, 24]. Their crystalline domains are especially resistant to deconstruction because the ordered arrangement of glucan chains promotes extensive hydrogen bonding within cellulose sheets, together with weaker hydrophobic interactions between adjacent sheets. At the molecular level, the chair conformation of the glucose residues positions the hydroxyl groups predominantly in equatorial orientations and the aliphatic hydrogen atoms in axial positions, thereby reinforcing interchain association and contributing to the dense hydrogen‐bond network shown in Figure 2c. This organization lowers the thermodynamic favorability of chain separation, restricts solvent penetration, and reduces access to glycosidic bonds. Hemicellulose is more structurally diverse and feedstock‐dependent, comprising xylans, mannans, glucomannans, and related heteropolysaccharides with variable branching and acetylation patterns. Rather than acting as an inert filler, hemicellulose serves as a dynamic interphase that coats fibril surfaces, modulates fibril aggregation, and mediates interactions between cellulose and lignin [21, 24, 72]. Its generally less ordered structure makes it more labile than crystalline cellulose, but its compositional diversity also contributes to feedstock‐specific differences in pretreatment response and downstream conversion behavior. Lignin adds a distinct dimension of complexity through its irregular aromatic network of guaiacyl, syringyl, and hydroxyphenyl units, which impart rigidity, hydrophobicity, and resistance to microbial attack while also shaping interfacial packing and deconstruction behavior [21, 25, 26]. These units arise from the primary monolignols coniferyl alcohol, sinapyl alcohol, and p‐coumaryl alcohol, respectively, and their relative abundance is a key determinant of lignin structural diversity (Figure 2d). Because lignin content and composition strongly influence biomass recalcitrance, lignin biosynthetic pathways have frequently been targeted to generate plants with reduced lignin levels or altered lignin chemistry. The hierarchical organization of lignocellulose should be viewed as a chemically heterogeneous and spatially structured composite in which tissue architecture, fibril organization, matrix composition, and interfacial packing jointly determine deconstructability.

Feedstock identity alone, however, is an insufficient predictive variable because substantial variability also occurs within a nominally identical biomass class. Ray et al. characterized 24 corn‐stover bales collected from four Iowa counties using 216 core samples and showed variability across field, bale, macro‐, micro‐, and molecular scales [73]. For Hardin and Story counties, combined glucan and xylan contents ranged from 50.4–62.6 wt% and 47.1–62.8 wt%, respectively, while lignin ranged from 14.3–20.8 wt% and 16.1–19.9 wt% [73]. These ranges illustrate why provenance, anatomical fraction, storage history, ash/mineral content, and analytical method should accompany bulk composition when data are used for transferable computational or ML models. The same principle applies across biomass classes: softwoods, hardwoods, grasses, and dicot stems differ in hemicellulose substitution, S/G/H composition, and lignin‐polysaccharide organization, so a structural model representative of one feedstock cannot automatically be assumed to represent another [21, 24, 25, 26, 72].

### 2.2 Molecular Origins of Recalcitrance

At the molecular scale, biomass recalcitrance arises from a convergence of stabilizing interactions rather than from a single dominant barrier. A major contributor is the hydrogen‐bonding network within and between cellulose chains, which promotes fibril cohesion and reduces the thermodynamic favorability of chain separation, swelling, and access to glycosidic bonds [10, 16]. In this sense, cellulose crystallinity is important not merely as a structural descriptor, but as a manifestation of the ordered hydrogen‐bonded and partially hydrophobic packing of glucan chains that limits accessibility to chemical and enzymatic attack. However, cellulose crystallinity alone is not a universal predictor of deconstructability. Kafle et al. monitored Avicel, bleached softwood, and bacterial cellulose during enzymatic hydrolysis and found that XRD crystallinity, degree of polymerization, and mesoscale packing did not consistently track the progressive decrease in hydrolysis rate [74]. In bleached softwood, the apparent crystallinity index increased from approximately 67% to 80% during the first 12 h while about 50% carbohydrate conversion had already occurred [74]. This behavior illustrates a central interpretive limitation: removal of amorphous hemicellulose or preferentially accessible material can increase the measured crystallinity index while overall digestibility improves. Crystallinity is therefore most useful as a relative descriptor within structurally comparable substrates and should be interpreted together with surface exposure, hydration, matrix composition, and accessibility. A second contributor is hemicellulose heterogeneity. Because hemicelluloses differ in backbone structure, substitution the extent to which the surrounding matrix is stabilized [24, 72]. Their branched and feedstock‐dependent nature means that pretreatment performance cannot be predicted reliably from cellulose crystallinity alone, since hemicellulose composition also shapes local hydration, interfacial association, and accessibility. A third, and often dominant, contributor is lignin, which acts not only as a hydrophobic barrier but also as a regulator of wetting, diffusion, interfacial association, and post‐pretreatment redistribution or condensation [25, 26]. Lignin therefore affects deconstruction both before and after pretreatment, limiting access to polysaccharides in the native wall and forming secondary barriers through migration, reprecipitation, or condensation during processing. Recalcitrance is thus better viewed as a coupled problem involving hydrogen‐bond stabilization, compositional heterogeneity, interfacial packing, and matrix shielding.

The relative importance of these descriptors changes with the limiting regime. In differently pretreated corn‐stover fractions, Liu et al. found that steam‐exploded material combined relatively low crystallinity with high porosity and showed favorable initial hydrolysis, whereas aqueous‐ammonia pretreatment produced the fastest hydrolysis and highest final glucan conversion through extensive lignin removal together with increased internal porosity [75]. The study concluded that lignin‐related effects were more important than crystallinity or porosity alone for the observed differences [75]. This does not establish lignin content as universally dominant; rather, it shows that a descriptor can lose predictive value after the bottleneck it represents has been relieved.

### 2.3 Inter‐Component Interactions and Accessibility Constraints

The strongest expression of biomass recalcitrance emerges at the interfaces between major wall components. Among these interfacial features, lignin‐carbohydrate complexes (LCCs) are particularly important because covalent motifs such as benzyl ethers, benzyl esters, and phenyl glycosidic linkages reinforce the integrity of the wall and increase the energetic cost of selective disassembly [22, 76]. Recent first‐principles studies further show that cleavage of benzyl ether LCC linkages can be kinetically accessible under acidic conditions yet remain thermodynamically unfavorable, implying that selective LCC deconstruction is strongly controlled by both pathway competition and process conditions [76]. Noncovalent interactions are equally consequential. Solid‐state NMR studies have shown that lignin forms extensive contacts with xylan‐rich regions rather than interacting uniformly with cellulose surfaces, and that these contacts vary across biomass classes and developmental stages [21, 25, 26, 72]. In mature Arabidopsis stems, for example, acetylated xylan preferentially associates with syringyl‐rich lignin, while denser carbohydrate‐lignin packing rather than lignin content alone appears to govern wall mechanics [26]. These observations shift the interpretation of recalcitrance away from bulk composition alone and toward the spatial distribution of interaction environments that actually govern accessibility during deconstruction. For this reason, accessibility is often a more operationally informative descriptor than composition alone when it is defined for a specific probe and processing environment. A biomass sample with moderate lignin content but poor pore connectivity and limited solvent penetration can remain less reactive than one with higher lignin content but more exposed cellulose surfaces and better transport pathways. This helps explain why simple correlations between bulk lignin percentage or nominal cellulose crystallinity and conversion efficiency often fail when extended across feedstocks or pretreatment chemistries [10, 16]. It also highlights a broader limitation of static descriptors: deconstruction depends on evolving accessibility under process conditions, including swelling, lignin migration, pore opening, and local restructuring. The central challenge is therefore not simply to disrupt biomass interactions, but to do so selectively and in a manner that improves downstream conversion without compromising carbon efficiency, product quality, or process sustainability.

Interfacial chemistry can also alter reaction energetics directly. Padmanathan et al. combined ab initio molecular dynamics, metadynamics, DFT, and thin‐film pyrolysis experiments to compare isolated cellobiose with benzyl‐ether LCC models [22]. Calculated activation barriers for transglycosylation and ring contraction were 60.98 and 68.15 kcal mol^−1^ for isolated cellobiose, but increased to approximately 108–110 and 113–117 kcal mol^−1^ in the corresponding LCC models [22]. This approximately twofold increase in activation barriers, together with three‐ to fourfold increases in reaction energies reported in the same study, provides quantitative evidence that covalent inter‐component structure can alter the reaction coordinate itself rather than merely acting as a passive transport barrier.

Quantitative studies also show that accessibility is conditional rather than universal. Grethlein reported a linear relationship between the initial enzymatic hydrolysis rate of acid‐pretreated hardwood and softwood and the pore volume accessible to a nominal 51 Å probe, whereas crystallinity index showed no corresponding relationship [77]. By contrast, Ishizawa et al. measured wet dilute‐acid‐pretreated corn stover by solute exclusion and ^1^H NMR thermoporometry and found no correlation between enzyme‐sized accessible pore volume and digestibility among samples giving approximately 70%–96% ethanol yields [78]. These results indicate that porosity is strongly predictive when transport is limiting but becomes less informative once sufficient pore opening has been achieved. Accessibility should therefore be reported as a probe‐, hydration‐, and method‐specific quantity rather than as a single intrinsic scalar property.

### 2.4 Structure‐Property Relationships and Implications for Modeling

The structural determinants of biomass recalcitrance cellulose packing, hemicellulose heterogeneity, lignin abundance and chemistry, LCCs, porosity, and accessibility define both resistance to deconstruction and the appropriate computational representation. Their predictive value is process dependent. Crystallinity and cellulose packing are most informative for intrinsic cellulose organization; lignin chemistry and spatial distribution become critical when shielding, adsorption, or condensation control conversion; porosity is most informative under transport‐limited conditions; and accessibility is the most integrative descriptor when it is defined for a specified solvent, enzyme, or catalyst environment. The literature therefore does not support a single universal recalcitrance index. A multidescriptor representation is more defensible than ranking all feedstocks by one bulk metric.

Model adequacy should likewise be property specific. Small cellulose or lignin fragments remain appropriate for local bond energetics, but they should not be interpreted as complete representations of fibril accessibility, lignin aggregation, solvent partitioning, or technical‐lignin behavior. Rather than prescribing a universal oligomer size, computational studies should report which structural feature is retained, demonstrate convergence where collective behavior is important, and validate the predicted observable against a corresponding experiment. Table 1 summarizes this descriptor‐to‐model‐to‐validation logic. The experimentally relevant comparison is therefore not whether one computational representation is globally realistic, but whether it preserves the physical feature controlling the target property.

**TABLE 1:** Structural determinants of lignocellulosic biomass recalcitrance: definition, experimental readouts, computational modeling approaches, predictive relevance, and key limitations.

Descriptor	Physical meaning	Experimental observables	Computational counterpart	Predictive value	Principal limitation
Cellulose crystallinity/packing [17, 34, 74, 79]	Degree of chain ordering and cooperative hydrogen‐bonding network	XRD, solid‐state ^13^C NMR, SFG	Periodic DFT, fibril models, atomistic MD	Informative within structurally comparable substrates	CrI from XRD is method‐dependent and may increase after removal of amorphous material
Bulk lignin content [16, 75, 80]	Overall abundance of the aromatic matrix	Klason lignin, compositional analysis	Composition‐constrained biomass models	Useful as a first‐order process descriptor	Does not reflect S/G/H ratio, condensation, distribution, or lignin–carbohydrate complexes (LCCs)
Lignin chemistry/distribution [21, 25, 26, 63]	Linkage types, monolignol ratios, and interfacial organization	2D‐HSQC NMR, thioacidolysis, GPC, Py‐GC/MS, ssNMR	DFT linkage models, representative lignin structures, MD interfaces	High mechanistic relevance for enzymatic and chemical deconstruction	Structural heterogeneity complicates the construction of unique, representative models
Porosity/pore connectivity [75, 77, 78]	Transport pathways through the cell wall	Solute exclusion, NMR thermoporometry, tomography, BET/BJH (where applicable)	Pore‐network models, CG‐MD, lattice Boltzmann, CFD	Strongly predictive when transport is rate‐limiting	Strongly dependent on hydration state, probe size, drying history, and pretreatment conditions
Accessibility [10, 77, 78]	Fraction of the structure reachable by a given probe	Enzyme‐accessible area, solvent uptake, swelling, probe exclusion, diffusion measurements	SASA, solvent occupancy, free‐volume and diffusion analysis	Integrative, process‐specific descriptor	Not a universal scalar; must be defined relative to a specific probe and environment.
LCC / interface chemistry [22, 63, 76, 81]	Covalent and non‐covalent interactions among wall polymers	NMR correlations, LCC isolation, chemical/enzymatic profiling	DFT, AIMD/metadynamics, explicit‐interface MD	Critical when interfacial chemistry governs cleavage or fractionation	Difficult to quantify comprehensively in native, unmodified biomass

### 2.5 Multiscale Nature of Recalcitrance and Design Implications

From a broader process perspective, biomass recalcitrance is intrinsically multiscale. Molecular interactions such as hydrogen bonding, aromatic association, and covalent cross‐linking propagate upward to shape fibril aggregation, pore architecture, solvent accessibility, and reactor‐scale conversion behavior, while temperature, residence time, solvent composition, and solids loading feed back onto the structure through swelling, lignin redistribution, carbohydrate stability, and mass transport [82, 83]. Recalcitrance is therefore a context‐dependent response of biomass structure to a deconstruction environment rather than a fixed material property.

This perspective also provides the transition to Chapter 3. Local covalent chemistry requires electronic‐structure methods; collective solvation and polymer organization require molecular dynamics; chemically evolving condensed phases require reactive simulation; pore and particle transport require mesoscale or continuum descriptions; and conversion rates and engineering performance require kinetic, reactor, process, TEA, and LCA models. In this Review, scale bridging refers specifically to the transfer of a defined and validated output, for example, an activation barrier, diffusion coefficient, solvent‐affinity descriptor, reduced kinetic mechanism, or particle response, into a receiving model. A model becomes decision relevant only when that handoff can change an experimental or engineering choice. The central challenge is consequently to preserve validity and uncertainty as information moves from idealized representations toward heterogeneous feedstocks and process conditions.

## 3 Computational Representations and Multiscale Modeling

**FIGURE 3:**
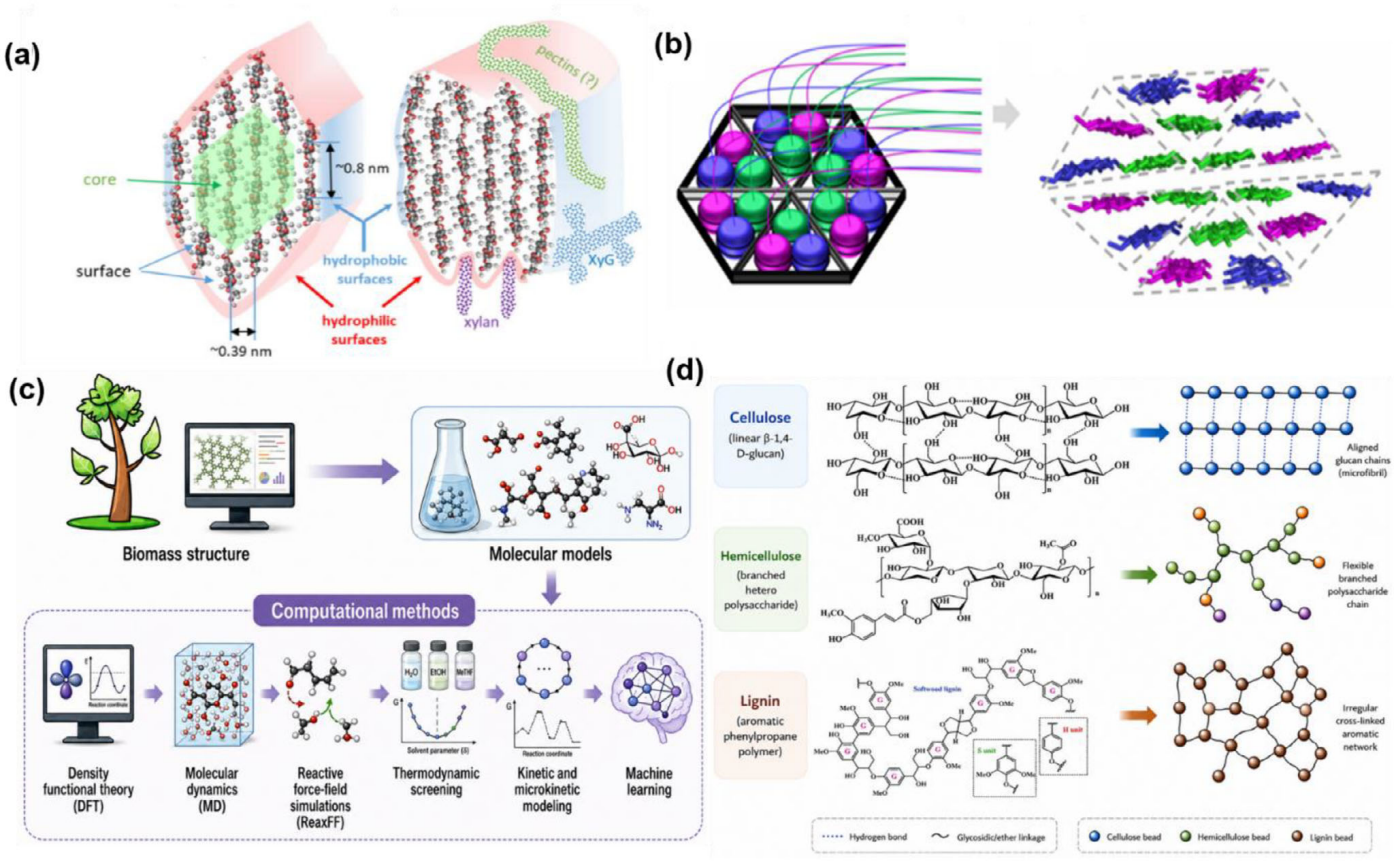
(a) Hierarchical cell‐wall architecture and cellulose‐microfibril organization; (b) biosynthetic origin and structural representation of plant cellulose microfibrils. Reproduced from [84] with permission from the American Chemical Society. Copyright 2024, American Chemical Society. Published under a Creative Commons CC BY 4.0 license. (c) Representative simulation routes for biomass dissolution, thermochemical conversion, and pyrolysis. (d) Representative atomistic and coarse‐grained descriptions of cellulose, hemicellulose, and lignin, illustrating the reduction of chemically explicit molecular structures to effective interaction sites for simulations over larger spatial and temporal scales.

The rational design of lignocellulosic biomass conversion increasingly depends on computational frameworks that resolve phenomena spanning electronic‐scale bond activation to reactor‐scale transport and selectivity. Yet predictive value depends less on methodological sophistication alone than on how biomass is represented at each stage of analysis. As summarized in Figure 3, computational representations form a hierarchy ranging from molecular fragments and oligomers to periodic surfaces, solvated condensed‐phase assemblies, reactive ensembles, and reduced process‐level models. Each representation captures a different aspect of biomass behavior, including intrinsic bond energetics, solvent‐mediated structural rearrangements, interfacial adsorption, reaction network evolution, and scale‐bridging workflow design. The central challenge is therefore not the availability of computational tools, but the need to align representation, method, and target observable so that mechanistic relevance is preserved across scales [10, 53]. This issue is particularly pronounced in lignocellulosic systems because the structural origins of recalcitrance are themselves multiscale. A model suitable for calculating the activation barrier of β‐O‐4 cleavage may be inadequate for describing lignin aggregation in mixed solvents. In contrast, a force‐field description that captures swelling or fibril association may not reliably resolve catalytic elementary steps. Accordingly, the field has moved beyond isolated single‐method studies toward integrated workflows in which quantum chemistry, molecular simulation, reactive dynamics, thermodynamic screening, kinetic analysis, and machine learning are deployed in a complementary manner [10, 53].

A useful organizing principle is therefore representation → calculation → experimental validation → uncertainty assessment → scale handoff. The methods discussed below should be viewed as complementary rather than competing, as each operates over a distinct spatial and temporal regime. Their value should therefore be assessed according to the specific observable they can reliably reproduce, the experimental or theoretical evidence used to validate that observable, and the physical meaning and uncertainty associated with the information transferred to the next scale.

### 3.1 Model Systems and Structural Representations

Accurate computational treatment of lignocellulosic biomass remains inherently challenging because biomass is chemically heterogeneous, conformationally flexible, and spatially organized across several orders of magnitude. Therefore, simplified model compounds remain indispensable in mechanistic analysis. Monomers and dimers are widely used to interrogate bond‐cleavage pathways, adsorption geometries, proton‐transfer steps, and catalyst‐substrate interactions because they permit higher‐level calculations and systematic comparison across catalyst classes [10, 53]. However, such representations only partially reflect the chemistry of native biomass. In cellulose, collective hydrogen‐bond networks and fibril packing can substantially alter accessibility and energetics relative to isolated chain fragments, whereas in lignin, oligomer length, linkage distribution, and conformational disorder strongly influence solvation, depolymerization, and recondensation behavior [9, 20]. Larger oligomeric and periodic representations are therefore required when the controlling question depends on collective interactions rather than intrinsic bond energetics alone. Cellulose is commonly represented as crystalline slabs, fibril fragments, or solvated assemblies to probe swelling, adsorption, and dissolution, whereas lignin is more often described using oligomer libraries or statistically representative structures that better capture realistic interunit linkages and monolignol distributions [85]. These choices are consequential. Assumptions such as the widespread use of the 18‐chain cellulose microfibril model in Figure 3b directly shape later predictions of exposed crystal faces, hydroxyl topology, solvent accessibility, and adsorption behavior [84]. The most informative representation is therefore not necessarily the most detailed one, but the one that preserves the structural feature that controls the phenomenon under investigation.

Model compounds should therefore be regarded as hypothesis‐specific representations. A lignin dimer can be suitable for linkage‐specific reaction energetics but cannot independently represent lignin aggregation or technical lignin molecular weight distributions; a short cellulose oligomer can probe local hydrogen bonding but not fibril‐scale accessibility. Three questions should accompany model construction: what observable is the model intended to reproduce, which controlling structural features are retained or omitted, and how can the consequences of that simplification be evaluated experimentally or through convergence analysis?

### 3.2 Electronic‐Structure Methods

Density functional theory (DFT) remains the leading electronic‐structure method for mechanistic studies in biomass valorization because it provides direct access to adsorption energetics, transition states, reaction intermediates, bond‐dissociation tendencies, and catalyst structure‐activity relationships [86, 87]. It has been widely used to study glycosidic bond cleavage, ring opening, dehydration, hydrogenation, hydrogenolysis, and hydrodeoxygenation pathways in both carbohydrate‐ and lignin‐derived substrates. Its particular strength lies in resolving elementary steps that are difficult to isolate experimentally, including competitive C–O versus C–C scission, proton‐coupled surface transformations, and the role of acid‐metal bifunctionality in tandem upgrading [88]. DFT‐derived descriptors also provide a critical foundation for kinetic, microkinetic, and machine‐learning workflows [58, 89]. Its limitations are equally clear. Most DFT studies still rely on relatively small model compounds, idealized surfaces, and static configurations, which restrict their ability to represent solvent‐induced restructuring, catalyst dynamics, and the conformational diversity of realistic biomass‐derived feeds. Solvent effects remain especially challenging: implicit models are often insufficient for capturing specific hydrogen bonding and competitive adsorption, whereas explicit solvation rapidly increases computational cost [35]. As summarized in Table 2, DFT is most powerful when its high‐fidelity outputs reaction energies, activation barriers, and adsorption descriptors are transferred to kinetic or microkinetic models rather than interpreted as complete predictors of macroscopic behavior.

**TABLE 2:** Comparative benchmarking and validation framework for computational methods in lignocellulosic biomass valorization.

Method	Primary scale	Defensible outputs	Experimental validation	Major uncertainty	Recommended handoff
DFT / electronic structure [28, 30, 31]	Local bonds, adsorbates, active sites	Reaction/adsorption energies; transition states; electronic descriptors; mechanistic trends	Kinetics; isotope effects; spectroscopy (surface/functional); product/selectivity trends	Functional dependence; static approximation; solvation/sampling limitations	Microkinetics; reactive‐potential training; ML descriptor generation
Classical MD [20, 36, 38]	Solvated polymers and interfaces	Conformations; RDFs; H‐bonds; diffusion; aggregation; accessible area	Density; scattering; NMR; swelling; solubility; transport data	Force‐field transferability; finite sampling; no chemical reactions	DFT structural ensembles; solvent analysis; mesoscale models
Reactive MD / ReaxFF [41, 46, 92]	Large chemically evolving systems	Reaction events; radicals; fragmentation; network motifs	Py‐GC/MS; TGA; gas evolution; product/char trends	Parameterization bias; accelerated heating; pathway/product overprediction	DFT refinement; network reduction; kinetic‐model construction
ML interatomic potentials [93]	Reactive atomistic systems	Near‐ab initio trajectories and reaction events	Independent ab initio benchmarks + experimental chemistry validation	Training‐set coverage; extrapolation in configuration space	Larger reactive simulations; network discovery
COSMO‐RS / screening [50, 51, 94]	Molecular solvent space; affinity/phase equilibrium	Activity coefficients; phase behavior; solubility ranking	Solubility; partitioning; pretreatment screening	Equilibrium assumption; transport/viscosity / reaction omitted	Down‐selection → MD/experiment → process screening
Kinetic/microkinetic models [58, 59, 91]	Reaction networks	Rates; selectivity; sensitivity; rate‐control analysis	Conversion/selectivity across temperature, time, and composition	Missing pathways; uncertain molecular parameters; deactivation/transport	Particle/reactor models; process simulation
Machine learning [39, 66, 95]	Descriptor/process latent space	Predictions; surrogates; screening; feature relationships	External / grouped test sets; prospective experiments	Data heterogeneity; leakage; extrapolation; weak uncertainty quantification	High‐throughput prioritization; fast reactor/process surrogates
CFD / reactor models [45, 59, 60]	Particle and reactor scale	Heat/mass transfer; residence time; conversion; yield	Reactor temperature; residence time; conversion/product distributions	Boundary conditions; closures; reduced chemistry	Process flowsheet; TEA/LCA; digital twin

The appropriate validation target should match the DFT claim. Proposed pathways can be tested against product distributions, isotope effects, spectroscopic data, reaction orders, apparent activation energies, or catalyst composition trends. Agreement with a single product observation is generally insufficient to establish a unique elementary mechanism, particularly when multiple pathways can generate similar macroscopic outcomes.

### 3.3 Molecular Dynamics Simulations

Molecular dynamics (MD) simulations complement DFT by resolving time‐dependent structural organization and condensed‐phase behavior that static electronic‐structure methods cannot capture. In lignocellulosic systems, MD is especially valuable for probing biomass‐solvent interactions, hydrogen‐bond disruption, cellulose swelling, lignin conformational dynamics, interfacial organization, and aggregation in mixed solvents [28, 36]. These capabilities make MD particularly important in pretreatment research, where performance is governed less by single‐bond energetics than by collective reorganization of biomass polymers in reactive media. Recent studies have used MD to clarify how solvent composition alters cellulose‐cellulose association, solvent‐accessible surface area, lignin condensation, and lignin redeposition under deconstruction conditions [37]. For example, MD analyses of acidic hydrotropic and deep eutectic solvent systems have shown that solvent composition can suppress excessive lignin condensation, promote lignin expansion, and shift the balance between lignin‐lignin and lignin‐solvent interactions in ways that directly affect delignification selectivity and preservation of native‐like lignin structure [90]. The strength of MD lies in its ability to represent realistic environments and evolving structures. Its limitations are structural rather than incidental. Classical MD is intrinsically nonreactive and cannot directly describe bond cleavage, repolymerization, or chemical conversion. At the same time, its reliability depends strongly on the quality of the force field and remains restricted to accessible time and length scales [40]. As indicated in Table 2, MD is most effective at generating structural ensembles, aggregation patterns, and transport descriptors that can be handed off to DFT, solvent‐screening analysis, or broader multiscale interpretation.

Two additional limitations deserve explicit attention. Force‐field transferability can be poor in highly concentrated ionic liquids, DESs, unusual protonation states, technical lignins, or high‐temperature environments, while slow aggregation, desorption, and phase restructuring may exceed accessible trajectories. Replicate trajectories, enhanced sampling, and comparison with density, diffusion, scattering/NMR, swelling, solubility, or other condensed‐phase measurements are therefore important. MD‐derived interaction energies should be interpreted as structural or transport descriptors rather than direct conversion metrics.

### 3.4 Reactive Simulations

When the problem shifts from structural reorganization to chemically evolving large‐scale systems, reactive force‐field methods such as ReaxFF become particularly valuable because they allow bond breaking and formation within a molecular dynamics framework [41, 42]. This capability has made reactive simulations especially important in biomass pyrolysis, thermal degradation, and reactive pretreatment, where reaction networks are too large and too dynamic to be predefined. In such systems, reactive MD can track the temporal evolution of intermediates, radicals, product fragments, and char precursors in simulations comprising thousands of atoms, thereby linking atomistic representation with emergent network behavior [43]. Its utility is strongest for comparing biomass components, evaluating temperature effects, and identifying dominant fragmentation motifs before kinetic reduction. These advantages are accompanied by substantial limitations. Reactive simulations are highly sensitive to parameterization, and force fields trained on restricted chemical spaces may perform poorly in systems containing carbohydrates, lignin, aromatic compounds, water, minerals, and reactive gases [44]. Predicted product distributions and dominant pathways can therefore vary with training scope, heating protocol, and system initialization. ReaxFF outputs should not be treated as direct experimental surrogates without calibration. As reflected in Table 2, their most robust role is as hypothesis‐generating tools whose outputs‐reaction events, radical evolution, and network motifs can be reduced into tractable kinetic schemes and subsequently tested against DFT, experiment, or reactor‐scale models [45, 64].

The accessible timescale also creates a thermal‐protocol mismatch. Reactive trajectories commonly require accelerated heating or elevated temperatures to generate sufficient chemistry within nanoseconds, which can alter pathway competition compared with laboratory pyrolysis. Accordingly, heating rate, density, trajectory length, system initialization, and species‐identification criteria should be reported when mechanistic conclusions depend on event frequencies.

### 3.5 Thermodynamic and Screening Methods

Alongside atomistic and reactive simulations, thermodynamic screening methods play a central role in solvent discovery and process design. Models such as COSMO‐RS are especially valuable because they enable rapid estimation of activity coefficients, solvation tendencies, and phase behavior from molecular structure and quantum‐chemical inputs, making them well‐suited to high‐throughput exploration of ionic liquids, deep eutectic solvents, and organic solvent mixtures [38, 49, 50]. In biomass pretreatment research, these methods have been widely used to rank candidate solvents for lignin extraction, cellulose dissolution, and multicomponent fractionation, thereby narrowing experimental search space and enabling more systematic solvent design [49, 50]. Recent studies illustrate this clearly: COSMO‐RS‐based screening of large ionic‐liquid libraries has identified strong lignin‐dissolving candidates and established hydrogen‐bond acceptor strength as a key determinant of lignin solubility, while computationally guided deep eutectic solvent design has shown how solvent selection can be coupled to targeted control of lignin linkage retention or cleavage during pretreatment [49, 50]. Yet the efficiency of thermodynamic screening also defines its main limitation. These methods are inherently equilibrium‐based and do not directly address kinetics, evolving biomass morphology, transport limitations, or degradation pathways, which often determine practical pretreatment performance. A solvent predicted to interact favorably with lignin may still fail in practice because of viscosity, competitive hydration, poor penetration, or solvent‐recovery penalties. As emphasized in Table 2, thermodynamic screening is most effective as a front‐end exploration tool whose outputs should be handed off to experiment and MD‐guided mechanistic interpretation rather than treated as complete process predictors [38, 49, 50].

### 3.6 Kinetic and Microkinetic Modeling

Kinetic modeling provides the critical link between molecular‐level insight and experimentally observable rates, selectivity, and product distributions. This link is essential in biomass valorization because even well‐resolved elementary chemistry does not, by itself, reveal which pathway will dominate under realistic conditions. By incorporating rate constants and mechanistic steps derived from DFT, experiments, or both, kinetic models enable identification of rate‐controlling steps, pathway competition, and the sensitivity of product distributions to temperature, catalyst loading, feed composition, and reactor environment [10, 45]. Microkinetic approaches are especially valuable in catalytic upgrading, where multiple elementary reactions proceed simultaneously on multifunctional or bifunctional surfaces. In contrast, reduced kinetic schemes remain indispensable in pyrolysis and other network‐rich systems where explicit mechanistic completeness is infeasible [57, 91]. Their application to biomass systems remains challenging because the underlying reaction networks are rarely complete. Real feeds are chemically diverse, catalyst surfaces may restructure or deactivate, and solvent effects may alter pathway accessibility. Model fidelity depends not only on whether the retained network captures the reactions that truly govern selectivity. Recent progress has accordingly relied on selective mechanistic reduction, in which quantum‐derived energetics, experimental constraints, and sensitivity analysis are combined to produce models that remain mechanistically informative while still tractable for process interpretation and scale‐up [8].

Uncertainty can propagate nonlinearly at this interface: an error of only a few kilojoules per mole in a rate‐controlling barrier can translate into orders‐of‐magnitude differences in the predicted rate, while omitted pathways, catalyst restructuring, deactivation, and transport can alter the apparent mechanism. Kinetic models should therefore be challenged across multiple temperatures, times, compositions, or reactor conditions rather than fitted to one conversion curve.

### 3.7 Machine Learning in Multiscale Workflows

Machine learning is increasingly incorporated into multiscale biomass modeling as both an accelerator and an integrative layer. Rather than replacing mechanistic methods, it is most effective when trained on outputs from DFT, MD, thermodynamic calculations, and experiments to predict properties or outcomes that would otherwise require expensive simulations or extensive empirical operations [10, 39, 53, 54]. In biomass valorization, such applications include catalyst screening, solvent ranking, lignin‐solubility prediction, pretreatment optimization, and surrogate modeling of reaction or process behavior [39, 53, 54]. Particularly promising are descriptor‐driven, graph‐based, and hybrid models that connect structural features with experimentally relevant targets such as dissolution performance, catalytic activity, and selectivity. The central requirement, however, is not predictive speed alone, but physical grounding. Models trained on narrow, heterogeneous, or weakly standardized datasets may appear accurate yet fail under extrapolation, a risk that is especially critical in biomass systems [39, 53, 54]. The strongest recent studies, therefore, combine interpretable descriptors, curated datasets, uncertainty awareness, and mechanistic priors. In this role, machine learning functions as a scale‐bridging technology: it compresses high‐dimensional outputs from lower‐scale models into design‐relevant surrogates, prioritizes regions of chemical space for detailed simulation, and reveals descriptor‐level structure‐property relationships that are difficult to extract through conventional analysis alone. As Table 2 indicates, its most effective downstream role lies in process optimization, design‐space exploration, and increasingly autonomous workflows.

For scale‐bridging applications, ML should report dataset provenance and chemical‐space coverage, grouped or external validation where feasible, uncertainty or applicability‐domain information, and performance under conditions that differ from the training distribution. Random splitting can substantially overestimate prospective performance when near‐duplicate conditions from the same feedstock, solvent, catalyst, or study occur in both the training and test subsets.

### 3.8 Method Integration and Scale Bridging

Table 2 shows that computational methods in biomass valorization are most powerful when coupled rather than compared. DFT provides chemically rigorous energetics; MD captures condensed‐phase structure and transport; reactive MD reveals evolving networks; COSMO‐RS screens solvent space; kinetic models connect mechanism to rates and selectivity; and ML enables surrogate prediction and accelerated design. Their accuracy is observable dependent: a method can be highly reliable for one quantity and inappropriate for another.

Validation should therefore be considered at four levels. Representation validation asks whether the model reproduces the material or environment being represented; mechanistic validation tests intermediates, trends, or kinetics; cross‐condition validation asks whether predictions survive changes in temperature, composition, or feedstock; and scale‐transfer validation asks whether the transferred quantity improves prediction of an independent observable at the receiving scale. These levels distinguish calibration from genuine predictive transfer and provide the basis for the integration framework developed in Chapter 8.

## 4 Solvent Selection, Pretreatment, Fractionation, and Recycle

**FIGURE 4:**
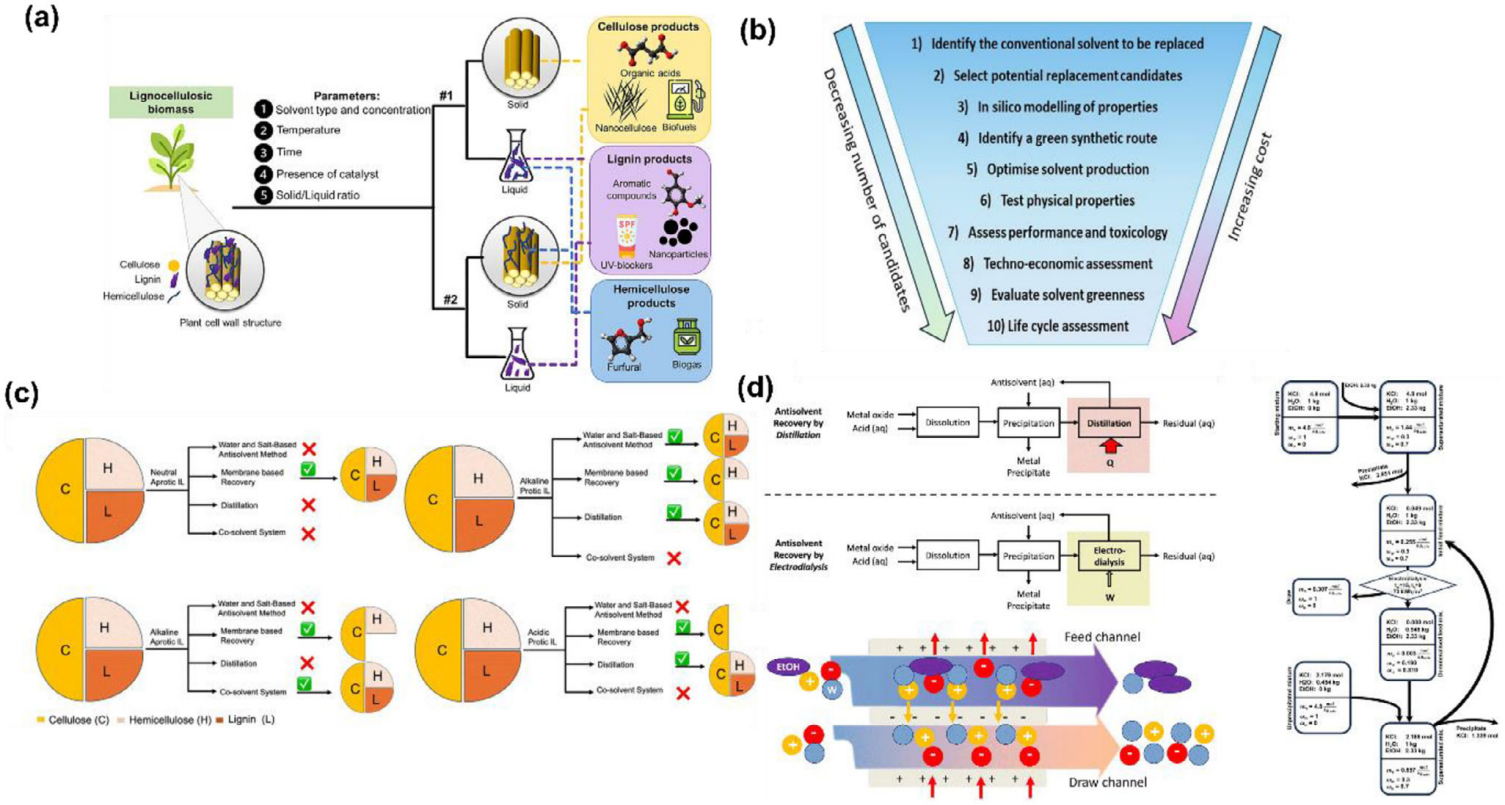
(a) Pretreatment and fractionation pathways directing cellulose, hemicellulose, and lignin streams. (b) Solvent‐selection pipeline combining in silico screening, physicochemical validation, and techno‐economic/life‐cycle assessment. (c) Recycling protocols for ionic‐liquid pretreatment. (d) Representative process configurations comparing distillation‐ and electrodialysis‐based antisolvent recovery for closed‐loop biorefinery integration. Panels (a,b) reproduced from Ref. [47] under the terms of the Creative Commons Attribution 4.0 International (CC BY 4.0) license. Copyright 2025, The Authors. Panels (c,d) reproduced from Ref. [48] under the terms of the Creative Commons Attribution 3.0 Unported (CC BY 3.0) license. Copyright 2025, The Authors.

Pretreatment is a defining upstream step in lignocellulosic biorefinery design because it controls not only cellulose and hemicellulose accessibility, but also the structure, purity, and downstream value of the lignin fraction. As summarized in Figure 4, pretreatment should no longer be viewed as an isolated dissolution or delignification step, but rather as an integrated workflow that links solvent selection, selective fractionation, product recovery, solvent recycle, and sustainability assessment [12, 47, 96, 97, 98, 99]. In this framework, the decisive metric is not deconstruction strength alone, but process‐specific fitness: the most effective solvent is the one that enables selective stream partitioning, practical recovery, manageable energy demand, and compatibility with downstream conversion. Figure 4 captures this logic by linking biomass partitioning (Figure 4a), staged solvent‐screening and validation (Figure 4b), solvent‐class‐dependent recycle strategy (Figure 4c), and recovery‐system design (Figure 4d) into a single decision architecture. The central implication is that solvent performance must be judged at the level of integrated process viability rather than isolated pretreatment severity.

Within the predictive workflow illustrated in Figure 1, solvent selection can be conceptualized as a sequential process of molecular candidate generation → thermodynamic screening → condensed‐phase mechanistic analysis → experimental pretreatment validation → product‐quality assessment → solvent recovery and recycle → TEA/LCA. The optimization objective should be defined by the intended product slate, because maximizing delignification, maximizing cellulose dissolution, and preserving valorization‐relevant lignin linkages represent distinct and potentially competing design targets.

### 4.1 Overview of Pretreatment Strategies

Lignocellulosic biomass pretreatment has evolved from simple solvent screening toward integrated solvent‐process design. Major solvent platforms include ionic liquids (ILs), deep eutectic solvents (DESs), organosolv media, hydrotropic and acidic aqueous systems, eutectic‐screening approaches, and biphasic solvent systems. Early studies established ILs as benchmark solvents for cellulose dissolution because of their strong disruption of cellulose hydrogen‐bonding networks [96]. Subsequent work broadened this perspective by emphasizing that effective pretreatment must be evaluated not only by dissolution ability, but also by fractionation selectivity, solvent recovery, process integration, and sustainability [47, 97]. Similarly, DESs emerged as tunable, low‐transition‐temperature solvent mixtures for lignocellulosic processing [98], but recent assessments caution that their “green” character depends strongly on recyclability, techno‐economic feasibility, and life‐cycle performance [99]. Organosolv systems remain among the most mature fractionation platforms, particularly for separating polysaccharides, lignin, and extractives, although scale‐up requires careful management of solvent recovery, safety, and cost [12]. Within this framework, Table 3 compares representative solvent systems according to their primary fractionation targets, advantages, limitations, and scale‐up trade‐offs. Aprotic ILs are highly effective for cellulose dissolution and cell‐wall disruption, but their industrial deployment is constrained by high viscosity, cost, water sensitivity, and demanding recovery requirements. Protic ILs offer improved potential for delignification and selective fractionation, although their stability and recyclability remain chemistry‐dependent. Cholinium‐based ILs provide a more biocompatible alternative, but still face challenges related to recovery and process economics [47, 48]. DESs occupy a complementary role. Their major strengths are low cost, compositional tunability, and compatibility with computational solvent design; however, their performance is highly formulation‐dependent and often limited by viscosity, regeneration efficiency, and reproducibility [49, 98, 99]. Organosolv systems provide a clear route to lignin‐first fractionation and high‐quality lignin streams, but solvent loss, flammability, and energy‐intensive recovery remain key barriers to large‐scale implementation [12, 100, 101]. Hydrotropic and acidic aqueous systems are attractive when preserving native‐like lignin structures is important, yet acid handling, neutralization, corrosion, and recycle complexity must be addressed [38]. More recent eutectic‐screening workflows and biphasic solvent platforms indicate a broader shift in the field: solvent selection is no longer based solely on extraction strength, but increasingly on separation efficiency, product quality, solvent recyclability, and downstream compatibility. Accordingly, pretreatment solvent choice should be viewed as a process‐specific optimization problem rather than a search for a universally superior solvent. An effective pretreatment strategy must therefore balance deconstruction efficiency, fractionation selectivity, solvent recovery, environmental burden, and techno‐economic viability within an integrated biorefinery context.

**TABLE 3:** Computational‐experimental decision matrix for pretreatment solvent platforms in lignocellulosic biomass valorization.

Solvent / platform	Primary computational role	Key experimental validation	Main strength	Prediction/process gap	Recovery/process concern
Aprotic ILs [36, 47, 48]	COSMO‐RS; MD of cellulose–ion interactions	Cellulose dissolution; swelling; sugar recovery	Strong disruption of cellulose structure	Affinity models do not capture viscosity, water sensitivity, or penetration behavior.	High recovery burden; solvent loss and cost
Protic ILs [47, 48]	Acid/base and thermodynamic screening	Delignification; carbohydrate retention; solvent stability	Tunable acidity and potentially simpler preparation	Proton‐transfer and reactive behavior can exceed equilibrium‐model predictions	Compositional stability during recycle
Cholinium ILs [47, 48]	MD; solubility / ML descriptors	Delignification and downstream biocompatibility	Favorable biocompatibility profile	Good affinity does not guarantee economically viable recovery	High water affinity and regeneration challenges
DESs [49, 51, 99]	COSMO‐RS; DFT/MD; ML	Lignin removal; β‐O‐4 retention; viscosity; sugar recovery	Large, tunable design space	Strong dependence on water content, composition, viscosity, and reactive side chemistry	HBA:HBD recovery and compositional drift
Organosolv [12, 100, 103]	MD/interfacial analysis; process modeling	Lignin quality; cellulose‐rich solids; solvent recovery	Mature fractionation logic; produces valuable lignin streams	Molecular‐scale effects do not capture flammability or distillation duty	Energy‐intensive distillation and solvent loss
Hydrotropic / acidic aqueous [38]	MD and reaction‐energy analysis	Lignin extraction; β‐O‐4 retention; carbohydrate recovery	High extraction selectivity under optimized conditions	Acid‐catalyzed chemistry and corrosion not represented by solvent models alone	Acid recycle, neutralization, and corrosion issues
Eutectic screening workflow [49, 51, 56]	High‐throughput COSMO‐RS / ML	Targeted testing of shortlisted candidates	Drastic reduction in experimental search space	Ranking can shift with viscosity, water content, kinetics, and real biomass heterogeneity	Use as a funnel for down‐selection, not final validation
Biphasic systems [104]	Phase‐equilibrium and partition modeling	Phase split; extraction yield; product distribution	Enables coupled reaction and separation	Multicomponent phase behavior exceeds simple screening models	Cross‐contamination and phase‐recovery challenges

### 4.2 Molecular‐Level Solvent‐Biomass Interactions

The effectiveness of pretreatment solvents is fundamentally governed by how they perturb the interaction network that stabilizes cellulose, hemicellulose, and lignin within the plant cell wall. Sarkar et al. provided an atomistic view of biomass recalcitrance in organosolv pretreatment. They showed, through molecular simulation, that polar protic solvents such as methanol and ethanol can separate lignin from cellulose irrespective of lignin charge, whereas aprotic solvents such as DMSO, THF, and 1,4‐dioxane preferentially separate uncharged lignin species [101]. In acidic hydrotropic systems, Zheng et al. used molecular dynamics to examine lignin extraction and condensation control in p‐toluenesulfonic‐acid‐based media, showing that the combined 1,4‐butanediol/pTsOH system could extract native‐like lignin under mild conditions with β‐O‐4 retention above 90% and lignin extraction above 80% [38]. These studies show that solvent efficiency cannot be reduced to a single bulk descriptor. Rather, it depends on the balance among hydrogen‐bond disruption, aromatic solvation, interfacial stabilization, and suppression of undesired recondensation pathways.

The strongest molecular studies therefore connect simulation outputs to measurable structural endpoints. Useful quantities include hydrogen‐bond populations, lignin radius of gyration, solvent‐accessible area, radial distribution functions, preferential solvation, and diffusion coefficients, but their relevance to the process should be established using swelling, solubility, viscosity, NMR‐derived linkage retention, fractionation yield, or enzymatic digestibility rather than interaction energy alone.

### 4.3 Computational Solvent Screening and Design

Computational methods have increasingly shifted solvent selection from empirical trial and error toward predictive design. Pontes et al. used a COSMO‐RS‐assisted workflow to screen 10 638 eutectic solvent combinations for lignin dissolution and laccase‐compatible operation, narrowed that space to 22 candidates for experimental testing, and identified acetamide:1,6‐hexanediol as an especially effective system with 46.5 wt% lignin solubility and 111% relative laccase activity [51]. In a complementary direction, Zhou et al. established a computational‐modeling‐guided DES design strategy to tailor lignin chemistry during lignocellulose pretreatment and showed that solvent composition can regulate β‐O‐4 cleavage versus preservation; in their study, choline chloride: lactic acid fully cleaved β‐O‐4 linkages, whereas other DESs better preserved lignin structure [49]. Machine learning has further accelerated this transition. Zhong et al. developed an explainable ML framework for lignin removal in DES‐based fractionation using a dataset of 467 pretreatment conditions; their best XGBoost model achieved R^2^ = 0.8259 in model development and R^2^ = 0.8034 on an independent validation set [56]. Zhang et al. extended this logic to lignin product quality following DES‐assisted hydrothermal treatment, showing that ML models could predict lignin yield, purity, and molecular weight distribution (Mw and Mn) with test R^2^ values of 0.78–0.97 [102]. These studies show that computational solvent design is moving beyond simple solubility ranking toward multivariate prediction of lignin quality, fractionation efficiency, and downstream suitability.

These successes should be interpreted within the domain that the screening method solves. COSMO‐RS and related equilibrium approaches do not directly represent wall penetration, viscosity‐controlled transport, evolving pore structure, solvent degradation, reactive lignin condensation, corrosion, or separation energy. Predicted rankings can also depend on how cellulose or lignin is represented, while water may simultaneously lower viscosity and alter the hydrogen‐bonding and ion‐association environment. A favorable calculated affinity is therefore a ranked hypothesis, not a complete pretreatment prediction.

A recent cellulose‐dissolution study illustrates a more stringent validation strategy. Zhao et al. assembled 329 solubility measurements spanning 195 ionic liquids, benchmarked COSMO‐RS, developed ML models, screened 1224 potential ILs, and experimentally evaluated five previously untested candidates [94]. This type of workflow is important because it exposes the mechanism‐based baseline and the data‐driven model to prospective experimental testing rather than relying only on retrospective agreement.

### 4.4 Fractionation Pathways and Selectivity

An effective pretreatment solvent must do more than dissolve biomass; it must separate lignocellulose into streams that retain high downstream value. Tofani et al. emphasized that organosolv treatment should be regarded as a resource‐based fractionation platform capable of extracting, separating, and isolating polysaccharides, lignin, and extractives while promoting selective depolymerization [12]. At the molecular scale, Sarkar et al. showed that solvent class strongly influences lignin‐cellulose separation behavior, helping to explain why certain organosolv formulations promote cleaner delignification than others [101]. In acidic hydrotropic systems, Zheng et al. demonstrated that carefully tuned solvent composition can recover structurally preserved lignin while maintaining favorable downstream processability of the carbohydrate‐rich residue [38]. Likewise, Zhou et al. showed that DES selection can control whether lignin linkages are preserved or deliberately cleaved during pretreatment, meaning that solvent design affects not only the amount of extracted lignin but also its structure and subsequent valorization route [49]. These findings reinforce that fractionation selectivity is not simply a matter of maximizing lignin removal. It requires tuning the solvent's acidity, polarity, hydrogen‐bonding capacity, and water content to extract lignin without excessive carbohydrate loss, lignin condensation, or a decline in cellulose quality. Figure 4a and Table 3 show that the choice of solvent dictates not only the extent of deconstruction but also the character and value of the resulting product streams.

Fractionation should consequently be treated as a multi‐objective problem. For lignin, the amount recovered should be evaluated alongside β‐O‐4 retention, condensation, molecular weight distribution, S/G/H composition, and residual carbohydrate. For the carbohydrate‐rich fraction, cellulose recovery, accessibility, hemicellulose retention or controlled solubilization, degradation‐product formation, and digestibility are relevant. Stronger pretreatment can improve one response while degrading another, so a single removal percentage should not rank a solvent.

### 4.5 Solvent Recovery and Recycling Challenges

Even when a solvent performs well during biomass deconstruction, its industrial relevance is often determined by recovery and recycle. Rahman et al. reviewed advances in ionic‐liquid recycling for lignocellulosic biomass pretreatment and concluded that large‐scale implementation depends on efficient and robust recovery strategies, as solvent loss, contamination, and regeneration costs remain central bottlenecks [48]. DES systems face analogous challenges. Contreras‐Gámez et al. emphasized that DES recycling, solvent stability, and scale‐up barriers must be discussed alongside pretreatment performance [99], while Verdía Barbará et al. likewise treated recovery and sustainability assessment as integral parts of solvent selection rather than downstream afterthoughts [47]. These studies support the interpretation in Figure 4c,d and Table 3 that solvent recovery is not peripheral but a primary driver of energy demand, cost, and viability. More critically, solvent and recycling choices are co‐design variables. A molecularly optimal solvent may fail given a specified recovery route, while a milder solvent can be superior if it enables a simpler, less energy‐intensive recycle loop.

Three recycle questions should be distinguished: recovery efficiency, chemical integrity of the recovered solvent, and reproducibility of pretreatment performance over repeated cycles. High gravimetric recovery can still conceal water accumulation, salt carryover, degradation products, or HBA:HBD compositional drift, while relatively small losses can dominate the cost or lifecycle impact for high‐embodied‐energy solvents. Recycle‐related descriptors should therefore enter candidate screening earlier rather than being treated only after solvent selection.

### 4.6 Process‐Level Considerations

The final selection of a pretreatment solvent system requires integrating molecular insights with process‐level decision tools such as techno‐economic analysis (TEA) and life‐cycle assessment (LCA). Contreras‐Gámez et al. explicitly reviewed the techno‐economic and environmental dimensions of DES‐based pretreatment and argued that scale‐up barriers cannot be resolved through solvent chemistry alone [99]. In a broader review of biorefinery optimization, Long et al. highlighted TEA and LCA as essential tools for connecting biomass deconstruction strategies with economically and environmentally viable scale‐up pathways [3]. Emerging primary studies reinforce this process‐level perspective. Wang et al. reported a prospective LCA of a scaled multicomponent DES pretreatment process. They found that, once solvent recovery was incorporated, the scaled system reduced environmental impacts by at least 67% across all midpoint categories relative to the laboratory‐scale case [13]. These studies show that a solvent cannot be considered optimal merely because it improves delignification or accessibility. A viable pretreatment platform must also tolerate recycle, limit energy‐intensive recovery, reduce emissions, and generate product streams whose value justifies the additional separation burden. Pretreatment is therefore not an isolated upstream unit operation, but the stage that conditions catalyst compatibility, product quality, and downstream conversion efficiency.

The scale handoff should also operate in reverse. If TEA or LCA identifies solvent recovery, water removal, or make‐up demand as the dominant hotspot, those variables should feed back into molecular screening so that the next design cycle penalizes candidates with unfavorable recovery characteristics. This feedback converts process assessment from a final pass/fail exercise into an upstream design constraint.

## 5 Catalytic Upgrading of Biomass‐Derived Intermediates

**FIGURE 5:**
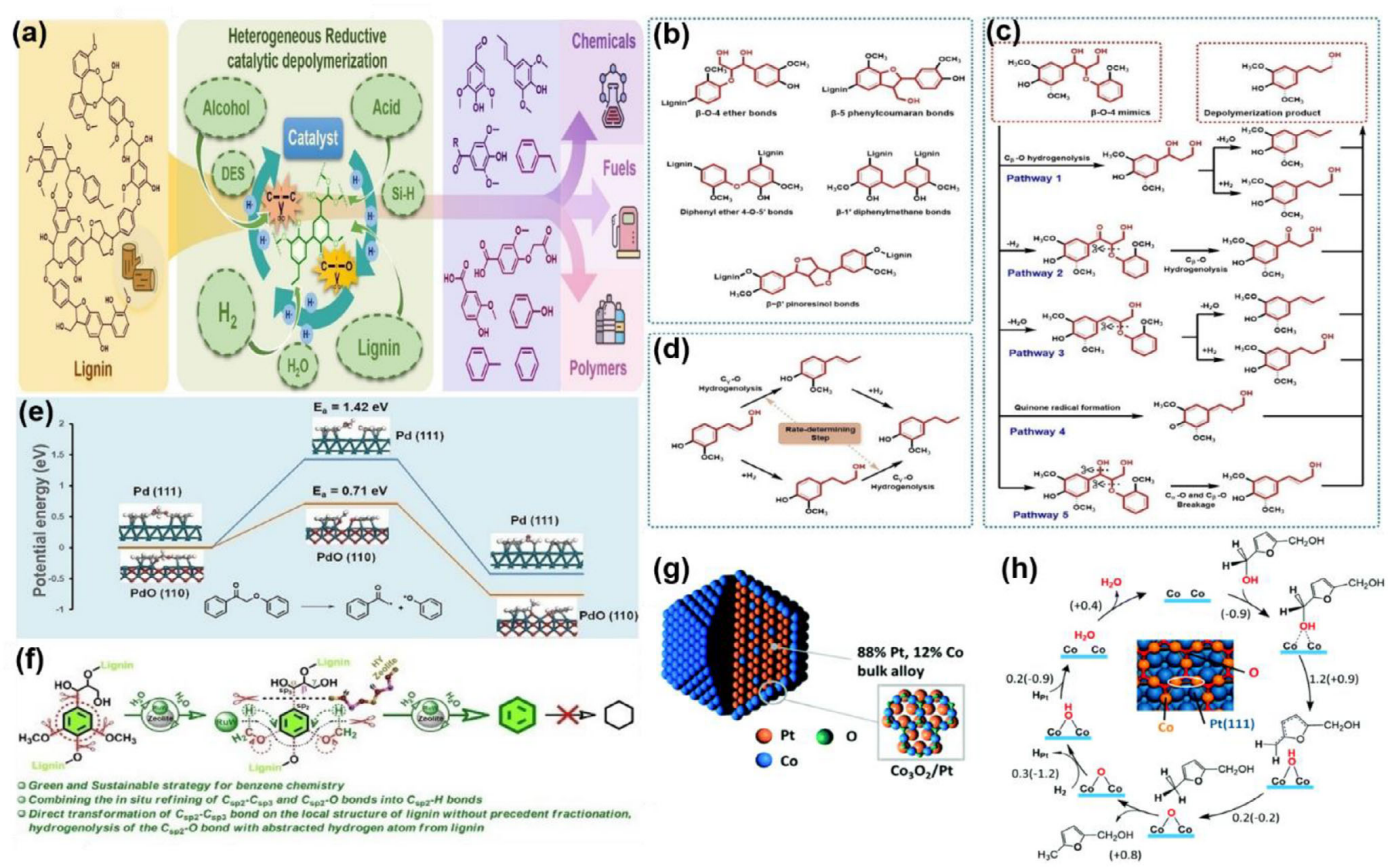
(a) Heterogeneous reductive depolymerization; (b) key lignin linkages; (c) β‐O‐4 hydrogenolysis; (d,e) 4‐propylguaiacol formation with DFT transition‐state energies for C–O scission; (f) HDO pathways for 5‐HMF and lignin to benzene conversion. (g) Pt_3_Co_2_ nanocrystal model involving an alloy core (88% Pt, 12% Co) covered with a Co_3_O_2_ surface oxide monolayer with a honeycomb structure. (h) Reaction mechanism of 2,5‐bis(hydroxymethyl)furan hydrodeoxygenation to 2‐hydroxymethyl‐5‐methylfuran on the Co_3_O_2_/Pt(111) surface. DFT reaction barriers (energies) are given in eV. The inset depicts a portion of a Co_3_O_2_/Pt(111) surface. Two Co atoms participating in C–O bond activation are encircled with a white ellipsoid. Panels (a‐f) reproduced from Ref. [105] with permission from Wiley‐VCH. Copyright 2025, Wiley‐VCH GmbH. Panels (g‐h) reproduced from Ref. [32] under the terms of the Creative Commons Attribution 3.0 Unported (CC BY 3.0) license. Copyright 2021, The Authors. Published by the Royal Society of Chemistry.

**FIGURE 6:**
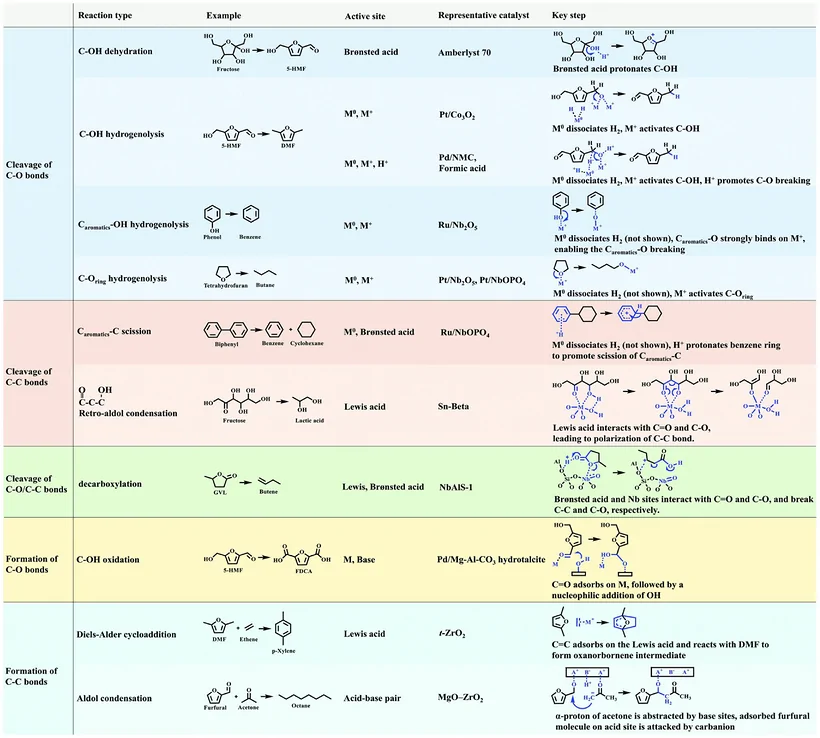
Comparison of bond‐scission and bond‐formation pathways, highlighting redox cycles involving reduced (M^0^) and oxidized (M^+^) metal centers, with acid‐base pairs (A^+^B^−^) stabilizing transition states and thereby influencing catalyst selectivity in biomass valorization. Reproduced from Ref. [32] under the terms of the Creative Commons Attribution 3.0 Unported (CC BY 3.0) license. Copyright 2021, The Authors. Published by the Royal Society of Chemistry.

Catalytic upgrading is a central step in lignocellulosic biorefineries because it converts highly oxygenated, often unstable intermediates into fuels, chemical precursors, and functional materials with improved energy density, stability, and end‐use value. Increasingly, biomass upgrading is treated as a multiscale problem in which elementary reaction chemistry, catalyst architecture, solvent effects, and reactor constraints must be considered together. In this framework, Figure 5 summarizes representative bond‐specific pathways and catalyst concepts for lignin‐ and carbohydrate‐derived intermediates, while Figure 6 provides a complementary reaction‐class and active‐site map linking C–O and C–C bond cleavage or formation to the catalytic functions that control selectivity.

Observed catalytic performance should therefore be interpreted as the integrated response of the catalyst‐solvent‐feed‐transport‐reactor system, rather than as a consequence of intrinsic reaction barriers alone. The relevant information pathway can be represented as molecular substrate → elementary reaction mechanism → active‐site architecture → experimental validation → reaction environment → catalyst‐particle and transport effects → reactor operation → catalyst lifetime and regeneration.

### 5.1 Carbohydrate‐Derived Platform Molecules

The upgrading of carbohydrate‐derived intermediates has received sustained attention because cellulose and hemicellulose‐derived sugars can be funneled into a relatively small set of strategically important platform molecules, including 5‐hydroxymethylfurfural (HMF), furfural, levulinate esters, sorbitol, and γ‐valerolactone (GVL). Román‐Leshkov et al. established the fructose → HMF → 2,5‐dimethylfuran (DMF) route and identified DMF as a biomass‐derived liquid‐fuel candidate with higher energy density than ethanol and no water miscibility [106]. More recently, Huang et al. reviewed direct catalytic conversion of cellulose and its derived sugars to HMF, levulinate esters, and sorbitol, emphasizing that catalyst selection must be matched to the full cascade of hydrolysis, isomerization, dehydration, hydrogenation, and esterification rather than to any single step [107]. Wang et al. similarly showed that acid‐base bifunctional catalysts can achieve very high HMF yields from fructose and glucose. At the same time, cellulose conversion remains more penalized by crystallinity and transport limitations [108]. Carbohydrate upgrading is therefore controlled by the coordination of multiple elementary steps rather than by a single catalytic function. Acid sites are required for hydrolysis and dehydration; Lewis‐acidic or Lewis‐basic environments facilitate glucose‐fructose interconversion; and hydrogenation becomes essential when the target slate includes sorbitol, DMF, or GVL. Complete performance depends on how effectively these functions are balanced while suppressing humins, rehydration, and over‐reduction. Figure 5 illustrates this principle through a bifunctional metal/oxide architecture in which hydrogen activation and C–O scission are spatially differentiated.

Carbohydrate upgrading is therefore a multiscale problem in its own right. Fructose to HMF or HMF to DMF performance cannot be transferred directly to glucose or cellulose because hydrolysis, isomerization, crystallinity, solid‐liquid transport, and unstable intermediates introduce additional constraints. Humin formation further narrows the selectivity window: under unfavorable acid‐catalyzed conditions, approximately 10–50 wt% of the initial substrate can be diverted into humin‐type products [109]. Product extraction, biphasic operation, short residence times, staged catalysts, or rapid downstream hydrogenation can consequently alter yield even when the intrinsic active site is unchanged.

### 5.2 Lignin Depolymerization and Valorization

Lignin valorization remains more demanding than carbohydrate upgrading because the substrate is chemically heterogeneous, structurally irregular, and highly prone to repolymerization once reactive fragments are released. Renders et al. described reductive catalytic fractionation (RCF) as a lignin‐first strategy that combines fractionation and catalytic stabilization, thereby suppressing repolymerization and generating low‐molecular‐weight lignin oils in close‐to‐theoretical monomer yield [110]. Li et al. later reviewed lignin‐first RCF and concluded that catalyst identity, solvent properties, lignin‐unit distribution, and stabilization timing jointly determine monomer yield, selectivity, and carbohydrate retention [111]. At the mechanistic level, Lu et al. showed that β‐O‐4 cleavage on Pd catalysts is governed not simply by bond strength, but by adsorption geometry and transition‐state stabilization, while Mensah et al. demonstrated that base‐catalyzed β‐O‐4 cleavage pathways depend strongly on local substitution pattern and on the competition between C–O and C–C scission routes [30, 31]. Efficient lignin upgrading, therefore, requires both bond cleavage and rapid stabilization of reactive phenolic intermediates. Catalysts that promote β‐O‐4 cleavage without adequate stabilization tend to broaden the product distribution through recondensation. In contrast, cleavage‐stabilization strategies, particularly lignin‐first approaches, improve monomer yield, product quality, separability, and downstream catalytic compatibility. This coupled logic is captured in Figure 5a, which frames heterogeneous reductive catalytic depolymerization as a system‐level interaction among catalyst, hydrogen donor, acidic medium, solvent environment, and lignin structure. Figure 5b further emphasizes that lignin depolymerization is bond selective: interunit motifs such as β‐O‐4, 4‐O‐5′, β‐1′, β‐5, and β‐β′ differ markedly in reactivity, which is one reason lignin upgrading remains governed by both linkage distribution and stabilization kinetics rather than by average lignin composition alone.

Lignin retains greater mechanistic emphasis in this Review because its representation problem is intrinsically broader: linkage distributions, S/G/H composition, polymer conformation, adsorption heterogeneity, technical‐lignin condensation, and fragment repolymerization all influence the observed network. This emphasis is therefore intended to reflect mechanistic complexity rather than greater economic importance relative to carbohydrate valorization.

### 5.3 Reaction Mechanisms, Catalyst Design, and Selectivity Control

Catalytic upgrading of lignocellulosic biomass proceeds through coupled elementary steps, including adsorption, proton transfer, bond activation, hydrogen addition or abstraction, intermediate rearrangement, and product desorption. These events are governed not only by intrinsic bond strengths, but also by substrate structure, catalyst surface chemistry, solvent environment, and feedstock‐derived impurities. In cellulose‐derived chemistry, cellulose‐to‐HMF conversion is commonly described through two principal pathways: an open‐chain route involving 3‐deoxy‐2‐ulose/3‐deoxyglucosone‐type intermediates, and a glucose‐fructose isomerization pathway followed by dehydration to HMF [107]. In lignin upgrading, however, bond dissociation energies alone are insufficient to predict reactivity because adsorption geometry, catalyst‐mediated transition‐state stabilization, and solvent‐mediated interfacial effects can redirect the reaction pathway [30, 112]. Thus, biomass upgrading mechanisms should be viewed as dynamic interfacial processes rather than isolated molecular transformations. Several catalyst‐design principles emerge from this mechanistic framework. First, multifunctionality is often indispensable: acid sites promote hydrolysis, dehydration, isomerization, rearrangement, and C‐C scission, whereas metal sites enable hydrogenation, hydrogenolysis, and hydrodeoxygenation. Second, metal‐support, metal‐oxide, and acid‐metal cooperation frequently determine selectivity more strongly than the identity of the metal alone. Third, deactivation resistance must be incorporated as a core design criterion, particularly under aqueous, lignin‐rich, acidic, or high‐temperature conditions where leaching, sintering, and coking are likely. For example, in HMF hydrogenolysis, interface engineering and cooperative active sites are critical for directing selectivity toward 2,5‐dimethylfuran (DMF) [113]. The mechanistic complexity of lignin upgrading is especially evident in β‐O‐4 cleavage chemistry. As summarized in Figure 5c, reported pathways include direct hydrogenolysis, Cα‐ketone‐ or enol ether‐mediated routes, radical pathways, and concerted C_α_‐O/C_β_‐O bond cleavage. These alternatives are not merely mechanistic variants; they explain why catalyst identity, hydrogen‐donor strength, solvent environment, and local lignin functionality can generate markedly different product distributions. Figure 5e,f further illustrate how selective lignin conversion can arise from Lewis‐acid/metal cooperation in side‐chain deoxygenation, mixed metal/oxide interfaces for C‐O activation and hydrogen transfer, and alloy‐acid bifunctionality for deeper deoxygenation toward simple aromatic hydrocarbons. XRD, XAS, and DFT calculations [29] reveal that the conversion of 5‐HMF to DMF over PtCo nanocrystals proceeds via a bifunctional mechanism, with the catalyst structure depicted in Figure 5g: a Pt‐rich core (88% Pt, 12% Co) responsible for H_2_ dissociation and a Co_3_O_2_ surface monolayer that drives C–O bond cleavage. The reaction is initiated by homolytic H_2_ splitting at Co/Pt sites, yielding a bridging Co–H–Pt intermediate. The dissociated H atoms subsequently reduce the carbonyl group of weakly adsorbed 5‐HMF to afford 2,5‐bis(hydroxymethyl)furan (BHMF). Next, as illustrated in Figure 5h, the Co_3_O_2_ coating catalyzes BHMF hydrodeoxygenation: two Co atoms mediate C–O bond scission, generating a loosely bound radical and an –OH group; the radical then abstracts hydrogen from the –OH group, forming 2‐hydroxylmethyl‐5‐methyl furan (HMMF) and a chemisorbed oxygen atom. A second C–O cleavage on HMMF produces DMF, while the chemisorbed oxygen reacts with H_2_ to form H_2_O, thereby closing the catalytic cycle. High selectivity for DMF is ensured because the Co_3_O_2_ surface preferentially promotes C–O scission while hindering furan‐ring chemisorption, thus effectively suppressing ring‐opening and decarboxylation side reactions.

More generally, lignocellulosic upgrading can be organized into four reaction classes: cleavage of C‐O bonds, cleavage of C‐C bonds, concurrent C‐O/C‐C cleavage, and formation of new C‐O or C‐C bonds (Figure 6). C‐O cleavage commonly proceeds through Brønsted‐acid‐catalyzed dehydration or metal‐catalyzed hydrogenolysis over M^0^/M^+^ sites. In selective hydrogenolysis, preserving C = C bonds is a major challenge, as seen in transformations such as HMF‐to‐DMF and phenol‐to‐benzene conversions. Catalyst architectures such as Pt/Co_3_O_4_ core‐shell structures, Pd/N‐doped mesoporous carbon with formic acid, and Ru/Nb_2_O_5_ have been used to tune hydrogen activation while suppressing undesired saturation. For ring C–O bond cleavage, strong Lewis acid sites coupled with efficient H_2_‐dissociating metals, such as Pt/Nb_2_O_5_, are particularly effective. C–C cleavage generally requires stronger acid functionality. Brønsted acids can promote β‐scission of aromatic side chains, whereas Lewis acids such as Sn‐Beta zeolites facilitate retro‐aldol condensation in carbohydrate‐derived intermediates. In reactions that require coupled intramolecular C–O/C–C cleavage, such as the conversion of γ‐valerolactone, close spatial cooperation between Brønsted and Lewis acid sites is essential. By contrast, C–O and C–C bond‐forming reactions are less common in biomass upgrading and typically involve –OH oxidation, Diels‐Alder chemistry for aromatic production such as p‐xylene, or aldol condensation; these processes often require basic rather than acidic or metallic sites. Thus, catalyst design for biomass upgrading must move beyond single‐site activity descriptors toward integrated control of adsorption, interfacial proton/electron transfer, hydrogen activation, bond scission, and product release. Because reaction pathways vary strongly with substrate, solvent, catalyst architecture, and operating conditions, rigorous mechanistic studies are essential for rational catalyst design and selectivity control.

Mechanistic claims should be validated at the same scale as the calculation. DFT can establish energetic plausibility, but stronger evidence requires consistency with experimentally observed intermediates or products, kinetic trends, isotope effects, reaction orders, or spectroscopy. Environmental robustness then requires that the mechanism remains valid when solvent, water content, substrate concentration, or feed complexity changes. Small molecules on idealized surfaces are therefore best interpreted as local mechanistic models rather than complete predictors of heterogeneous‐feed behavior.

### 5.4 Reaction‐Network Complexity and Reaction Environment

The interconnected nature of biomass upgrading chemistry means that selectivity is a network property rather than a single‐step property. Desired pathways are continually competing with rehydration, polymerization, over‐hydrogenation, coke formation, and humin formation. Velasco Calderón et al. examined humin formation mechanistically and reported that 10–50 wt% of the initial substrate can be diverted into humins under unfavorable conditions [109]. de Jong et al. later reviewed industrial humins and showed that humin formation is not a marginal side issue but a central problem of selectivity, carbon efficiency, and processability in carbohydrate upgrading [114]. Under these conditions, a catalyst that accelerates dehydration may still lower isolated HMF yield if it simultaneously promotes humin growth or rehydration. At the same time, stronger hydrogenation activity can improve intermediate stabilization but also increase the risk of over‐reduction. Catalyst performance is likewise conditioned by the reaction environment, especially solvent identity, temperature, hydrogen pressure, water content, and the presence of impurities or reactive intermediates. As emphasized by Dong et al., solvent effects in heterogeneous biomass catalysis should be understood as dynamic interfacial phenomena rather than merely as bulk‐medium effects [112]. A catalyst that performs well for a model compound in a dry organic solvent may therefore behave very differently in aqueous, lignin‐rich, salt‐containing, or solids‐bearing feeds. Biomass upgrading is thus fundamentally a catalyst‐solvent‐feed‐reactor problem.

These competing pathways also make reactor configuration part of selectivity control. Broad residence‐time distributions can increase secondary rehydration, condensation, or over‐hydrogenation, while pore diffusion can cause large lignin‐derived oligomers to react preferentially near external surfaces. In gas‐liquid‐solid systems, nominal hydrogen pressure does not uniquely determine the concentration experienced by a catalyst particle because gas‐liquid transfer, solvent solubility, wetting, and agitation intervene. Reactor‐scale transport should therefore be considered when comparing intrinsic kinetic differences.

### 5.5 Emerging Data‐Driven Catalyst Design

Recent advances in machine learning and catalyst informatics are opening new routes for catalyst discovery and optimization in biomass valorization. Zheng et al. reported a machine‐learning‐assisted optimization strategy for polyoxometalate‐catalyzed lignin oxidation and depolymerization. They showed that incorporating virtual sample generation and prior knowledge improved the average R^2^ from 0.66 to 0.98, demonstrating that data‐driven workflows can substantially accelerate catalyst and condition selection for lignin upgrading [115]. More broadly, Parveen and Slater showed that machine learning becomes most powerful when paired with structured metadata, high‐throughput experimentation, design‐of‐experiments logic, and interpretable tools such as SHAP analysis, particularly for catalytic production of bio‐based chemicals [61]. In this context, ML serves less as a replacement for mechanistic interpretation than as an integrative layer that links quantum descriptors, catalyst formulation variables, solvent effects, and process parameters. Its greatest value lies in accelerating hypothesis generation and design‐space exploration while remaining grounded in mechanistic and operando evidence.

Catalysis‐focused ML should also include substrate identity, solvent and reaction‐condition metadata, catalyst synthesis history, and deactivation or time‐on‐stream information where available. A high retrospective R^2^ should not be interpreted as evidence of transfer from model compounds to technical lignins, from batch to flow operation, or from one catalyst family to another without grouped or prospective validation.

### 5.6 Challenges in Industrial Translation

Despite major mechanistic advances, a substantial translational gap persists between model compound studies and practical biomass upgrading. Real lignocellulosic feedstocks are chemically heterogeneous and batch‐variable, and they often contain ash and inorganic impurities that alter reaction kinetics, catalyst stability, and downstream separations. Reviews of woody‐biomass inorganics show that alkali and alkaline‐earth species can affect preprocessing and thermochemical conversion through fouling, corrosion, and catalyst deactivation [116]. In parallel, lignin‐focused studies increasingly emphasize that technical lignins differ markedly from native model structures because isolation and pretreatment frequently generate more condensed, less β‐O‐4‐rich, and more chemically diverse substrates, complicating direct transfer of mechanistic insights from idealized dimers or oligomers to process‐relevant feeds [117]. Continuous‐flow lignin depolymerization studies further show that feedstock pretreatment and catalyst deactivation remain central engineering constraints even when promising catalytic activity is observed at the laboratory scale [62]. The catalytic‐upgrading literature, therefore, no longer asks simply whether biomass‐derived intermediates can be converted, but whether they can be converted selectively and stably under process‐relevant conditions. Carbohydrate upgrading depends on controlling tandem acid/base/metal sequences while suppressing humins and overreaction, whereas lignin valorization depends on cleavage‐stabilization strategies that prevent repolymerization as fragments are formed. The next stage of catalyst development will consequently rely on tighter integration of DFT, operando mechanistic analysis, solvent‐aware reaction engineering, machine‐learning‐guided optimization, and complexity‐aware feedstock modeling.

Industrial translation, therefore, requires more than merely demonstrating high initial activity. A meaningful assessment of catalyst stability should include activity and selectivity as a function of time‐on‐stream, catalyst mass balance, metal leaching, coke or deposit formation, regeneration behavior, repeated‐cycle performance, and post‐reaction characterization. A useful progression toward process relevance is single model compound → representative mixture → isolated technical fraction → real biomass‐derived stream → continuous operation. Changes in catalyst ranking along this sequence should be examined systematically, as they may reveal the effects of competitive adsorption, transport limitations, catalyst poisoning, or reaction‐network complexity, rather than being dismissed as incidental discrepancies.

## 6 Pyrolysis and Reaction‐Network Modeling

**FIGURE 7:**
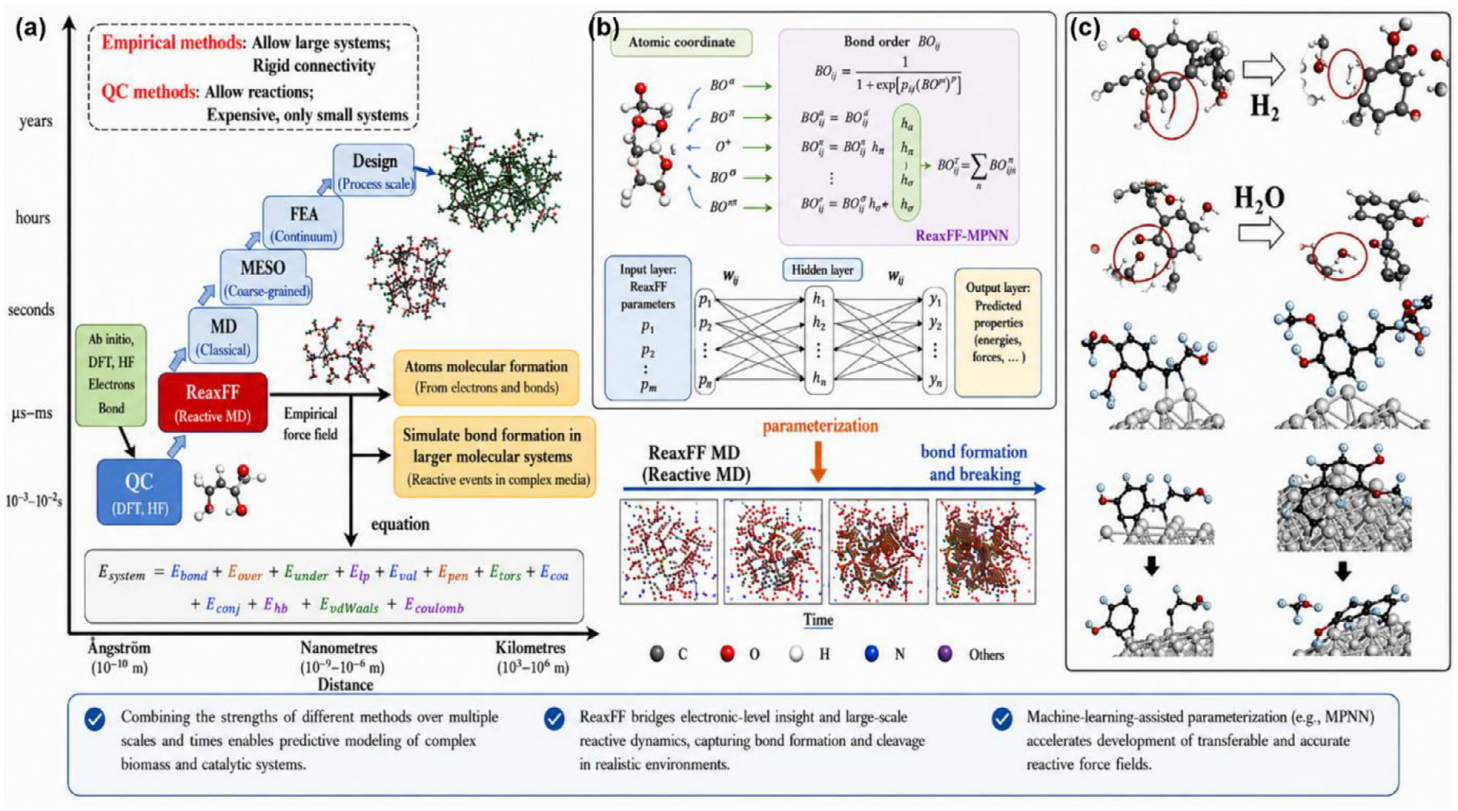
Multiscale framework for reactive molecular modeling using ReaxFF. (a) Relative spatial and temporal domains of quantum‐chemical, reactive MD, classical MD, coarse‐grained, continuum, and process‐level approaches, highlighting ReaxFF's role in bridging electronic‐scale chemistry with larger reactive systems. (b) Conceptual ReaxFF framework showing bond‐order‐dependent interactions, force‐field parameterization, ML‐assisted parameter optimization, and reactive‐MD simulation of bond formation and cleavage. (c) Representative chemical transformations accessible to reactive force‐field simulations, including hydrogenation, hydration, condensation/esterification, and bond scission. Adapted from Ref. [92] with permission from Elsevier. Copyright 2022, Elsevier.

Pyrolysis remains one of the most consequential thermochemical routes for lignocellulosic biomass valorization because it converts solid biomass directly into bio‐oil, permanent gases, and char without requiring prior isolation of molecularly defined intermediates [118, 119]. Unlike catalytic upgrading of relatively well‐resolved platform molecules, pyrolysis proceeds through nonequilibrium reaction cascades in which bond cleavage, radical generation, rearrangement, volatilization, cracking, and condensation unfold over strongly overlapping time and length scales. Product distributions therefore depend not only on nominal temperature, but also on biomass composition, ash content, heating rate, and vapor residence time [118, 119]. Hemicellulose, cellulose, and lignin decompose over partially distinct temperature windows and generate chemically different product families, which explains the marked feedstock sensitivity of pyrolysis behavior [118, 119]. Figure 7 summarizes the multiscale position of ReaxFF, its bond‐order and parameterization framework, and representative bond‐forming and bond‐cleaving transformations relevant to reactive biomass simulations [92].

Pyrolysis therefore provides a particularly clear test of multiscale integration, following the sequence feedstock structure → elementary chemistry → reactive‐network discovery → kinetic reduction → particle‐scale transport → surrogate modeling → reactor‐scale CFD → product recovery and process evaluation. Because information can be simplified or lost at each transition, validation should be performed independently at the molecular, kinetic, particle, and reactor scales rather than inferred from agreement at only one level.

### 6.1 Molecular Level Reaction Mechanisms

At the molecular scale, pyrolysis is initiated by cleavage of the covalent motifs that define biomass polymer architecture, followed by rapid rearrangement and fragmentation of highly reactive intermediates. For cellulose, Wang et al. combined Py‐GC/MS with DFT and showed that the major fast‐pyrolysis products include levoglucosan, glycolaldehyde, and 5‐hydroxymethylfurfural, and demonstrated that selectivity arises from competition among parallel pathways such as transglycosylation, fragmentation, furan formation, and dehydration [120]. However, native biomass chemistry cannot be reliably inferred from purified cellulose models alone. Padmanathan et al. showed, through a combination of ab initio molecular dynamics, metadynamics, DFT, and thin‐film pyrolysis experiments, that lignin‐carbohydrate complex linkages substantially perturb cellulose decomposition, with cross‐linked cellobiose exhibiting approximately twofold higher activation barriers and three‐ to fourfold higher reaction energies than isolated cellobiose [22]. For lignin, current mechanistic understanding consistently supports a staged description involving initial bond cleavage, primary radical evolution, and subsequent charring and deoxygenation reactions [121]. Accordingly, DFT is most valuable in pyrolysis research when used to resolve linkage specific energetics and discriminate among competing elementary pathways.

### 6.2 Reactive Molecular Dynamics and Machine‐Learned Potential Fields

**FIGURE 8:**
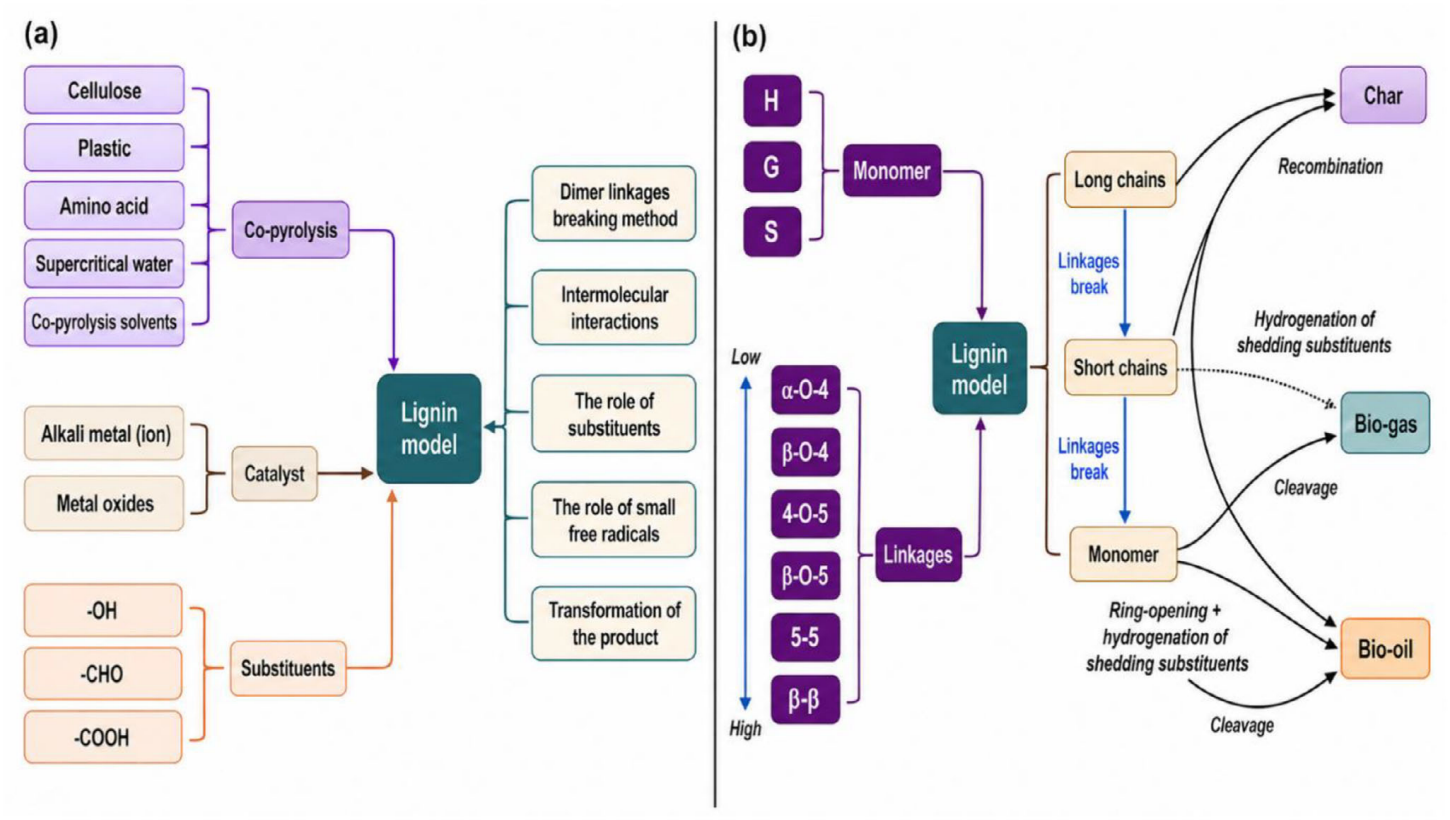
Conceptual framework for modeling lignin pyrolysis and co‐pyrolysis. (a) Principal variables influencing computational lignin‐pyrolysis models, including co‐pyrolysis partners, catalysts, substituent chemistry, intermolecular interactions, linkage‐breaking mechanisms, radical chemistry, and product transformation. (b) Relationship between lignin monomer composition and interunit‐linkage distribution, and their evolution through long‐chain, short‐chain, and monomeric intermediates toward char, bio‐oil, and permanent gases. Adapted from Ref. [64] with permission from Elsevier. Copyright 2026, Elsevier.

Reactive molecular dynamics, particularly ReaxFF, has become an important tool in biomass pyrolysis research because it can explicitly capture bond‐breaking and bond‐forming events in systems that are too large and chemically complex for direct DFT simulations [46, 93, 122]. As shown in Figure 7, ReaxFF occupies an intermediate position between quantum‐chemical methods and conventional empirical force fields. Unlike classical force fields, it uses bond‐order‐dependent energy terms to describe chemical reactivity, while retaining sufficient computational efficiency for large, dynamically evolving systems [46, 93, 122]. This capability is especially valuable in biomass pyrolysis, where the number of possible decomposition, recombination, and secondary transformation pathways rapidly exceeds what can be captured using predefined reaction networks [46, 122]. ReaxFF simulations of simplified biomass assemblies containing cellulose, hemicellulose, and lignin have shown that these components generate overlapping gas product distributions, while still exhibiting distinct dominant decomposition pathways and activation trends [6]. More recent studies on hardwood lignin pyrolysis have further demonstrated ReaxFF's ability to track β‐O‐4 cleavage, functional‐group removal, volatile formation, char evolution, and apparent kinetic behavior within a unified reactive framework [46]. The mechanistic breadth of this approach is reflected in Figure 8a, which summarizes major directions in lignin‐pyrolysis modeling, including model complexity, catalytic effects, substituent chemistry, radical pathways, intermolecular interactions, and product transformation [46, 121, 122]. Similarly, the reaction network in Figure 8b illustrates how lignin structures evolve through chain scission, linkage cleavage, recombination, ring opening, side‐chain hydrogenation, and secondary fragmentation, ultimately distributing carbon among char, bio‐oil, and gas products [46, 122]. The principal strength of reactive MD is therefore not only its ability to reproduce individual elementary steps, but also its capacity to reveal competing reaction channels without imposing a predefined mechanism. This makes it particularly useful for network discovery, mechanistic hypothesis generation, and the identification of dominant transformation motifs under pyrolytic conditions. However, conventional reactive force fields still face quantitative limitations, especially when small energy differences determine pathway selectivity, radical stability, or product branching. These limitations have stimulated the development of ab initio deep‐learning potential fields for cellulose and biomass pyrolysis, which aim to combine near‐DFT accuracy with the length and time‐scale advantages of molecular dynamics [93]. Accordingly, ReaxFF and machine‐learned potentials should be regarded as complementary tools rather than replacements for electronic‐structure calculations or experiments. Reactive MD is most powerful for exploring complex reaction networks and identifying plausible mechanisms, whereas key energetic trends, rate‐controlling steps, and selectivity predictions require validation against experiment or higher‐level quantum‐chemical benchmarks [46, 93, 122]. In advanced biomass‐pyrolysis modeling, the most rigorous strategy is therefore a hierarchical workflow: reactive MD for pathway discovery, DFT or ab initio calculations for energetic refinement, and experimental measurements for validation of product distributions, kinetics, and char evolution.

Reactive MD should nevertheless be interpreted primarily as a network‐discovery method. Empirical reactive force fields are sensitive to parameterization, while accelerated heating and short accessible trajectories can alter branching relative to experiments. Species and reaction count also depend on bond‐order thresholds and analysis rules. Replicate trajectories, higher‐level energetic checks, and comparison with Py‐GC/MS, TGA, gas evolution, or char/product trends are therefore required before event frequencies are treated quantitatively.

### 6.3 Reaction‐Network Construction, Reduction, and Kinetic Modeling

Biomass pyrolysis can involve hundreds to thousands of elementary events; therefore, explicit construction of reaction networks and systematic reduction are indispensable for any model intended for particle‐ or reactor‐scale prediction [59]. Gao et al. assessed the CRECK biomass‐pyrolysis kinetic framework in multiscale simulations and demonstrated that a 32‐reaction, 58‐species scheme preserves meaningful feedstock sensitivity while remaining tractable for coupling with particle and reactor models [59]. This explains why lumped and semi‐detailed kinetic models remain central to pyrolysis engineering: they are not chemically exhaustive, but they retain the dominant sensitivities required for scale‐up. Their value depends less on mechanistic completeness than on whether they capture the observables that control volatile release, tar cracking, bio‐oil yield, gas composition, and char formation under reactor‐relevant transport conditions [59]. Figure 8b emphasizes that lignin pyrolysis is inherently a branching network rather than a simple sequence of independent bond‐cleavage events [46, 122]. Long chains can recombine and contribute to char formation; short fragments can continue to crack; and monomeric products can either stabilize into bio‐oil components or evolve further into gas‐phase species. Network construction and reduction are therefore not merely numerical conveniences, they are necessary steps for translating reactive complexity into models that remain interpretable at larger scales [46, 59, 122].

A reduced mechanism should consequently be judged by whether it preserves the higher‐scale observable of interest, for example, volatile‐release rate, gas/condensable/char partitioning, major product classes, or feedstock sensitivity, rather than by the number of elementary reactions retained. More detailed chemistry is not automatically more predictive if poorly constrained parameters introduce larger uncertainty or prevent practical reactor coupling.

### 6.4 Machine Learning for Network Discovery, Surrogate Modeling, and Downstream Thermodynamics

Machine learning is increasingly used to address the combinatorial challenges of pyrolysis network discovery, kinetic fitting, and product‐yield prediction [55, 65, 95, 123, 124, 125]. A recent comprehensive review concluded that random forests, XGBoost, and artificial neural networks already achieve strong predictive performance for pyrolysis yields, while explainability tools such as SHAP and partial dependence analysis are beginning to connect model outputs to process‐level interpretations [65]. Representative primary studies support this view. Cheenkachorn et al. compiled 273 experiments from 38 literature sources and showed that XGBoost outperformed alternative models for simultaneous prediction of bio‐oil, bio‐char, and gas yields, achieving training R^2^ values above 0.90 while identifying chemically meaningful sensitivities to cellulose, hemicellulose, lignin, temperature, and heating rate [123]. At a more mechanistic level, Ji et al. introduced a chemical reaction neural network (CRNN) framework that learned interpretable reaction pathways and kinetic parameters directly from TGA data without prior expert specification of the network [55]. More recent extensions have shown that CRNN‐based schemes can also incorporate inorganic effects and secondary reactions during cellulose pyrolysis [124]. However, data quality remains a major constraint. Pascarella et al. assembled a standardized dataset of 1137 records from 160 sources and showed that model performance and explainable AI (XAI)‐derived interpretation remain highly sensitive to data harmonization, missing‐value handling, and curation strategy [95]. Within this broader workflow, COSMO‐RS is not a primary tool for describing initial bond breaking, but it is increasingly useful downstream for bio‐oil phase behavior, solvent screening, and the recovery of value‐added compounds from the aqueous bio‐oil fraction [125]. The most promising role of ML in pyrolysis, therefore, lies not in replacing mechanistic models but in accelerating network discovery, compressing multiscale outputs, and guiding downstream separation and valorization decisions [55, 65, 95, 123, 124, 125].

The data problem is correspondingly more specific than a lack of record count. Literature datasets often omit or inconsistently report reactor geometry, vapor residence time, heating‐rate definition, particle size, ash/inorganic composition, moisture, pretreatment history, collection efficiency, and analytical uncertainty. These omissions create uncertainty in experimental, representational, model, and distribution shifts. Grouped or external validation is therefore particularly important for pyrolysis ML because random splitting can place nearly identical conditions from the same study in both training and test sets.

### 6.5 Reactor‐Scale Modeling and Process Implications

From an engineering perspective, the decisive challenge is coupling molecular and kinetic descriptions to the reactor scale, where particle size, intraparticle heat transfer, residence time, and vapor‐phase secondary reactions strongly reshape final product distributions [59, 60, 126, 127]. Lu et al. addressed this issue by coupling detailed kinetics, an ML‐derived intraparticle model, and CFD for fluidized‐bed fast pyrolysis, achieving an average bio‐oil yield prediction error of 6.4% across four feedstocks [60]. Catalytic fast pyrolysis adds another layer of complexity because catalysts restructure the vapor‐phase network by promoting deoxygenation, cracking, aromatization, and coke formation [59, 60, 126, 127]. Recent reviews identify zeolites, carbonaceous catalysts, and metal oxides as the principal catalyst classes controlling bio‐oil composition and aromatic formation [128]. Within zeolitic systems, hierarchical micro/mesoporous ZSM‐5 composites have been shown to tune aromatic selectivity and coke behavior [126], while phosphorus‐modified ZSM‐5 shifts selectivity away from coke and light gases toward liquid bio‐oil, yielding an 11% relative increase in carbon yield from biomass to aviation fuel together with a 14% relative decrease in minimum fuel selling price [127]. El Bari et al. place pyrolysis within an integrated biorefinery framework rather than treating bio‐oil, gas, and char as isolated outputs [129]. In their findings, pyrolysis gas can be upgraded through gasification and downstream catalytic routes such as methanol synthesis, methanation, or Fischer‐Tropsch conversion; bio‐oil can be fractionated and upgraded through hydrogenation, hydrodeoxygenation, extraction, or physical refining; and biochar can be converted into activated carbon, modified materials, or catalyst and catalyst‐support platforms for subsequent upgrading steps. This systems view reinforces a broader conclusion from the pyrolysis literature: the most valuable models are not those that merely predict primary product distributions, but those that connect molecular‐ and network‐scale chemistry to downstream valorization, circular process integration, and system‐level sustainability [59, 60, 127]. The pyrolysis literature supports three conclusions. First, pyrolysis must be treated as a reaction‐network problem rather than a single thermochemical event. Second, the most informative mechanistic studies are those that couple bond‐level chemistry to evolving condensed‐phase structure and transport. Third, the most credible predictive frameworks are hybrid, combining first‐principles chemistry, reactive dynamics, reduced kinetics, machine‐learning surrogates, and reactor‐aware modeling within an integrated multiscale workflow [60, 65, 93].

The Lu et al. framework is one of the clearest current examples of validated adjacent‐scale coupling [60]. Detailed kinetics were embedded in an intraparticle reaction/transport model; the computationally expensive particle response was compressed using an ML surrogate; and the surrogate was coupled to fluidized‐bed CFD. Validation across four feedstocks yielded an average bio‐oil‐yield prediction error of approximately 6.4% [60]. The significance of this example is the explicit information handoff and reactor‐level validation; its limitation is that the final prediction still inherits uncertainty from the reduced kinetics, particle representation, surrogate training domain, feedstock characterization, and CFD closures. Agreement at the reactor scale therefore validates the coupled workflow for the tested observable and domain without proving that every lower‐scale reaction pathway is uniquely correct.

## 7 Machine Learning Integration in Biomass Valorization

**FIGURE 9:**
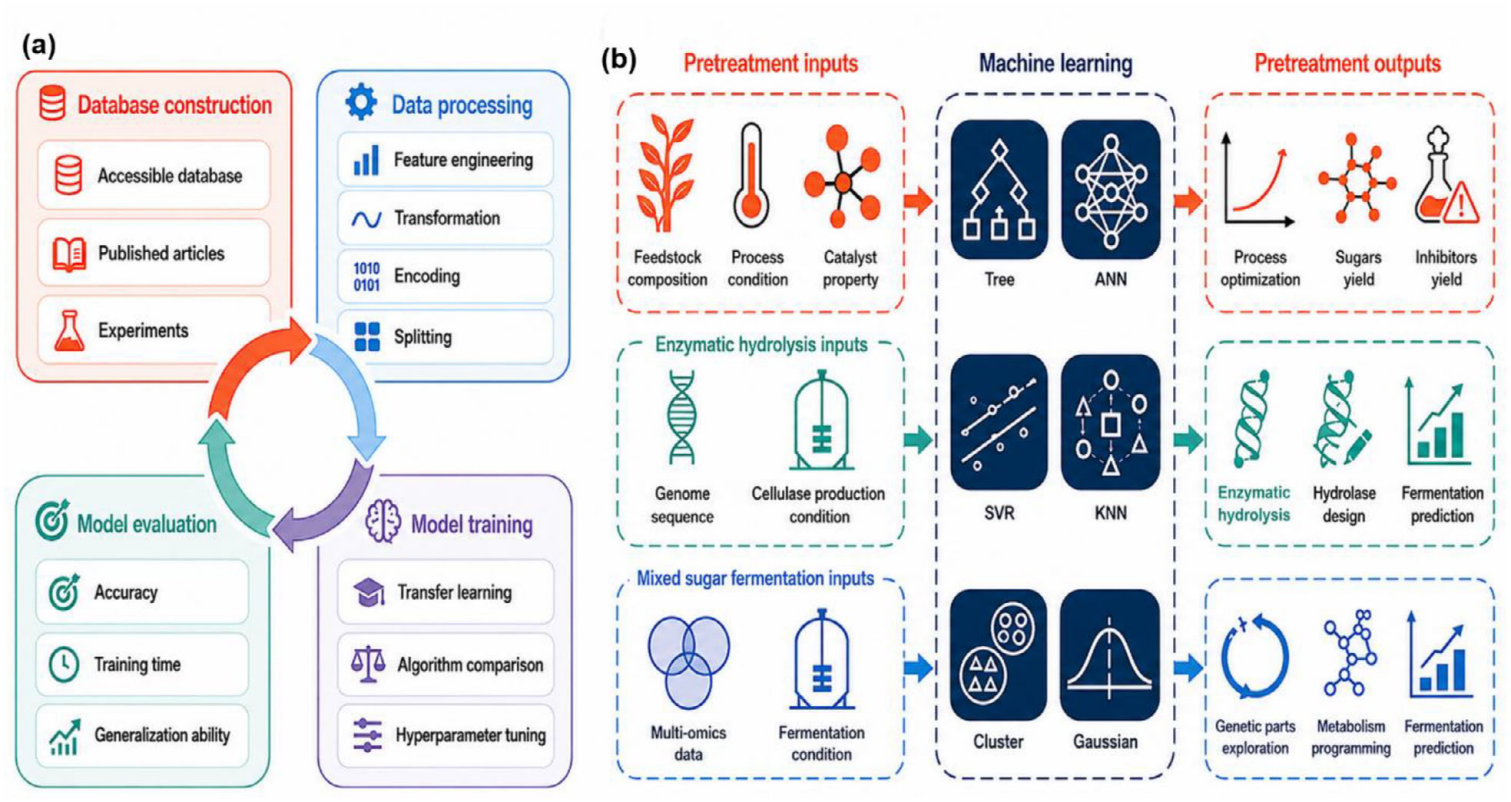
Machine‐learning workflow and representative applications in lignocellulosic biomass valorization. (a) General ML‐development cycle comprising database construction, data preprocessing and feature engineering, model training and hyper‐parameter optimization, and evaluation of predictive accuracy, training efficiency, and generalization. (b) Representative ML applications across pretreatment, enzymatic hydrolysis, and mixed‐sugar fermentation, integrating feedstock, process, catalyst, genomic, multi‐omics, and fermentation descriptors with tree‐based models, artificial neural networks (ANN), support vector regression (SVR), k‐nearest‐neighbor (KNN), clustering, and probabilistic methods. Adapted from Ref. [39] with permission from Elsevier. Copyright 2025, Elsevier.

**FIGURE 10:**
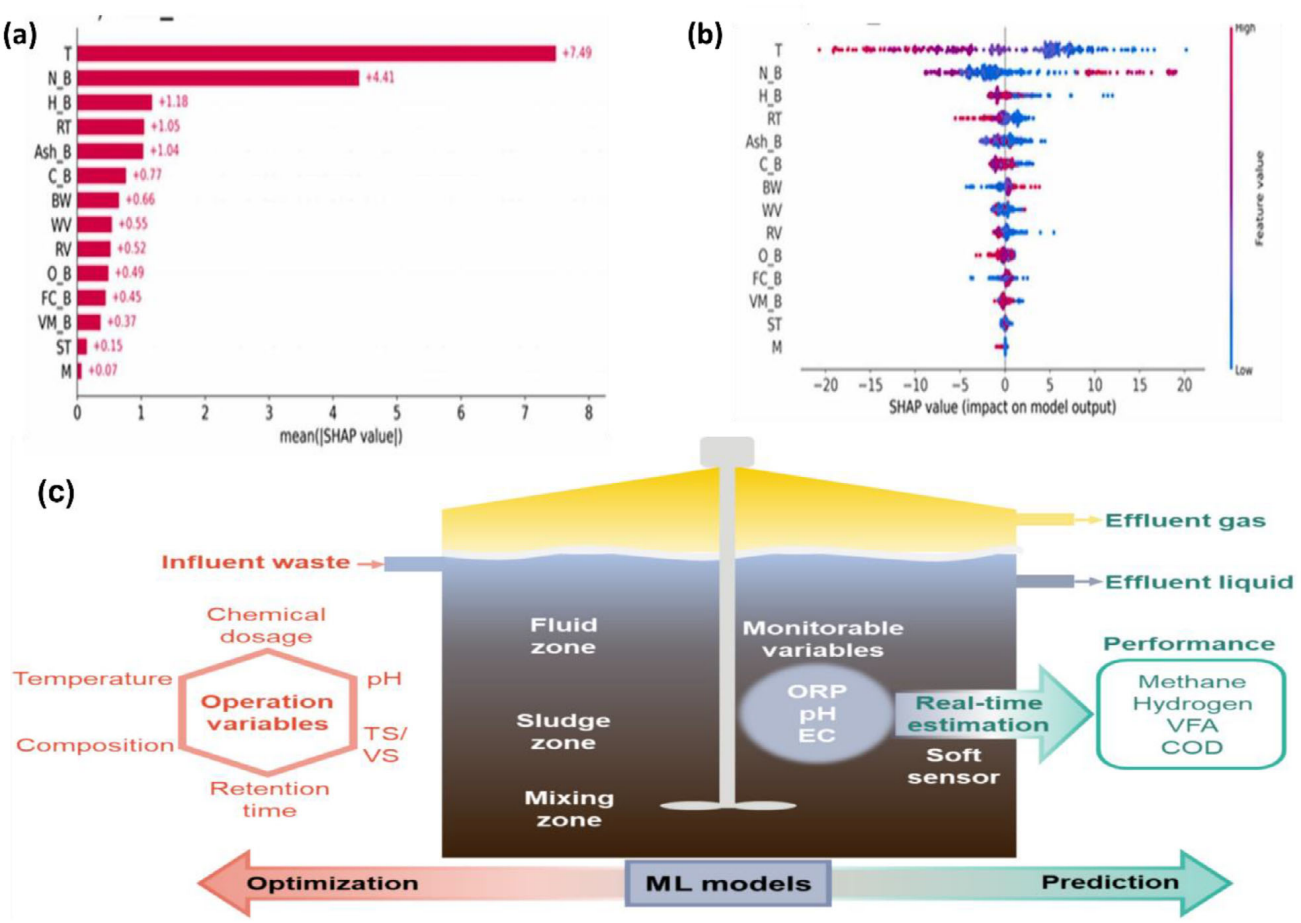
Explainable machine‐learning approaches for identifying influential variables and supporting process optimization in biomass conversion. (a) Global feature‐importance ranking for hydrochar yield; (b) SHAP analysis showing the direction and distribution of individual feature contributions to predicted hydrochar yield; and (c) machine‐learning‐assisted anaerobic digestion enabling performance prediction, process optimization, and real‐time monitoring. Panels (a,b) adapted from [133] under the Creative Commons Attribution 4.0 International Licence (CC BY 4.0). 2025 The Authors, published by Elsevier Ltd. Panel (c) reproduced from Ref. [39] with permission from Elsevier. Copyright 2025, Elsevier.

Machine learning is becoming a unifying framework for biomass valorization, not merely as a tool for isolated prediction, but as a connector among experiments, molecular modeling, and process design within a common decision framework. As shown in Figure 9, this role spans structured model development from curated datasets (Figure 9a) and applications across pretreatment, enzymatic hydrolysis, and mixed‐sugar fermentation (Figure 9b); and deployment in soft sensing, real‐time estimation, and digital bioprocess control (Figure 9c). Interpretability is considered separately in Figure 10, which illustrates SHAP‐based model explanation and feature‐importance/F‐score analysis as tools for identifying dominant predictors while preserving the distinction between statistical importance and mechanistic causality. In this context, ML should be viewed less as a stand‐alone screening tool than as an integrative framework that renders heterogeneous datasets, simulations, and process models interoperable across the biomass value chain. The central implication is that the value of ML lies not simply in predictive accuracy, but in its ability to compress multiscale complexity into interpretable, uncertainty‐aware, decision‐relevant workflows for biorefinery optimization.

The scope of this chapter is methodological. It addresses how ML models are represented, trained, validated, interpreted, and bounded by uncertainty and domain of applicability. FAIR repositories, ontologies, metadata, APIs, provenance, and digital operational infrastructure are treated separately in Chapter 9.

### 7.1 Roles of Machine Learning Across the Value Chain

ML is now applied across nearly every stage of biomass valorization, although the maturity of these applications differs substantially by process stage. As summarized in Figure 9b, current uses span pretreatment, enzymatic hydrolysis, and fermentation‐oriented process optimization, but predictive robustness and industrial maturity remain uneven. In a pretreatment study, Zhong et al. developed an explainable ML framework for DES‐based lignin fractionation using a dataset of 467 pretreatment conditions, demonstrating that combining solvent descriptors with operating variables can predict lignin removal with both high accuracy and mechanistic interpretability [56]. In biphasic pretreatment, Madadi et al. showed that gradient‐boosted regression successfully optimizes cellulose preservation, hemicellulose removal, and delignification across a broad operating space [104]. For downstream hydrolysis, Xie and Fan demonstrated that support vector regression outperformed several alternative models for predicting enzymatic sugar release across different pretreatments, highlighting the continued strength of classical supervised models when datasets are moderately sized but rich in structured descriptors [130]. In thermochemical conversion, ML is increasingly used to predict pyrolysis yields, rank process variables, and support reactor‐condition optimization. However, recent reviews emphasize that pyrolysis remains especially sensitive to reactor variability and dataset heterogeneity [65, 95]. The literature therefore suggests that pretreatment and solvent design are currently the most mature ML application domains; hydrolysis modeling is becoming increasingly predictive when pretreatment history and substrate descriptors are available; and pyrolysis ML is advancing rapidly but remains more dependent on careful data harmonization.

The maturity of these applications should be distinguished from their reported accuracy. Pretreatment optimization and pyrolysis‐yield prediction now contain relatively substantial literature datasets, whereas catalyst‐family transfer, kinetic‐network learning, and online biomass‐process control remain more limited. The strongest evidence is prospective: a model should ultimately identify a solvent, catalyst, feedstock, or condition not represented in training and be challenged by a new experiment.

### 7.2 Data, Algorithms, and Interpretability in Biomass ML

The impact of machine learning (ML) in biomass valorization is determined first by the quality, completeness, and chemical relevance of the underlying data. A persistent bottleneck is the lack of comprehensive, standardized databases that combine structural, thermodynamic, catalytic, feedstock, and process information across length scales [54]. Although ML models can outperform empirical correlations in tasks such as predicting biomass higher heating value from proximate and ultimate analyses [131], their reliability depends strongly on the descriptors used to encode the system. In biomass valorization, variables such as catalyst composition, solvent identity, feedstock origin, pretreatment severity, reaction conditions, and characterization metadata all influence model transferability. Feature engineering is therefore a chemically consequential step rather than a routine preprocessing task. Molecular fingerprints, solvent descriptors, DFT‐derived parameters, process‐history variables, and compositional features each capture different physical information and impose different limits on extrapolation. As emphasized in Figure 9a, database construction, cleaning, and representation are themselves scientific challenges, not merely technical steps before modeling. A broad spectrum of algorithms has been applied to biomass conversion, including linear regression, decision trees, random forests, support vector machines, gradient boosting, artificial neural networks, and increasingly, structure‐aware and hybrid models [39, 54, 132]. However, model choice should be dictated less by algorithmic complexity than by dataset size, data quality, target property, and the need for interpretability. For example, support vector regression has been shown to perform strongly on relatively modest but well‐structured pretreatment and hydrolysis datasets [130]. Similarly, catalysis‐focused ML studies indicate that generalizability, computational efficiency, descriptor quality, and interpretability are often more important than model sophistication alone [132]. Pyrolysis studies further demonstrate that predictive accuracy and mechanistic interpretation are highly sensitive to missing‐data treatment, dataset curation, and standardization protocols [65, 95]. A practical division of roles is therefore emerging. Classical supervised‐learning models remain effective for medium‐sized tabular datasets in pretreatment, hydrolysis, and process optimization. Gradient‐boosting methods are particularly useful when nonlinear feature interactions and variable ranking are important. Higher‐capacity neural networks and structure‐aware models become more attractive when molecular representation, catalyst structure, or reaction‐network complexity is central. Hybrid and knowledge‐guided ML approaches are especially valuable when physical consistency, mechanistic plausibility, and extrapolative reliability are more important than interpolation accuracy. Interpretability is essential for converting ML predictions into chemical insight. Figure 10a,b illustrates how complementary feature‐importance and SHAP analyses can be used to interrogate machine‐learning predictions in biomass conversion. In the hydrothermal‐carbonization study of Abdeldayem et al. [133], feature‐importance analysis and SHAP‐based interpretation identified biomass and process variables as influential predictors of hydrochar properties, with process temperature emerging as a particularly important determinant of hydrochar yield. The agreement between global feature‐importance measures and SHAP‐based attribution provides a more informative assessment of model behaviour than either metric alone, while also helping to identify variables that warrant mechanistic and experimental investigation. Importantly, such associations should be interpreted as model‐derived relationships rather than evidence of causality; their physical meaning depends on the representativeness of the training data, feature correlations, and independent validation. Thus, ML in biomass valorization should be developed as a data and chemistry‐informed workflow rather than as a purely algorithmic exercise. Studies should report data provenance, descriptor rationale, missing‐data treatment, validation strategy, uncertainty, and interpretability analysis. Without these elements, even accurate models may remain difficult to generalize; with them, ML can become a powerful tool for connecting feedstock complexity, catalyst design, solvent effects, process conditions, and product selectivity.

Data scarcity in biomass ML has at least four forms: numerical scarcity, chemical‐space scarcity, process‐space scarcity, and metadata scarcity. A dataset may contain hundreds of rows yet only a small number of chemically independent solvents, catalysts, or feedstocks. Likewise, multiple temperatures for the same formulation increase record count without providing the same information as additional chemical families. Missing provenance, ash, water content, recycle state, reactor geometry, catalyst aging, or analytical uncertainty can be as limiting as a small sample size.

Validation strategy should therefore match the intended deployment. Leave‐solvent‐out, leave‐feedstock‐out, leave‐catalyst‐family‐out, leave‐study‐out, temporal, and prospective validation provide progressively stronger tests of transfer than random record splitting. An ML prediction should also be accompanied by information on whether the new point lies within the interpolation domain, near its boundary, or outside the chemical/process region represented in training. Descriptor distance, ensemble disagreement, Gaussian‐process uncertainty, conformal intervals, or related methods can provide useful domain and uncertainty indicators.

Interpretability should likewise be used cautiously. SHAP values and feature‐importance scores explain how a trained model uses its inputs; they do not establish causality. Figure 10 therefore illustrates these methods as sources of testable hypotheses rather than as mechanistic proof. Mechanistic conclusions should be strengthened using DFT, MD, spectroscopy, kinetics, or targeted perturbation experiments.

### 7.3 Integration With Computational Chemistry and Multiscale Modeling

One of the most productive roles of ML in biomass valorization is its integration with computational chemistry. Zhong et al. explicitly coupled computational‐chemistry‐derived solvent descriptors with ML to accelerate DES screening for lignocellulosic pretreatment [56]]. More generally, Jiang et al. framed ML‐assisted biomass valorization around three connected functions: basic‐data generation, process modeling, and system optimization, thereby establishing a natural interface between simulation outputs and data‐driven prediction [54]. From the perspective of catalytic biomass conversion, Parveen and Slater likewise argued that digitalization depends on the structured integration of catalyst data, reaction metadata, and chemically meaningful descriptors, so that ML models learn from electronically and chemically informed inputs rather than from disconnected empirical variables alone [61]. This framework is directly relevant to descriptors derived from DFT, MD, COSMO‐RS, kinetic modeling, and related simulations, particularly when such descriptors are used to encode solvent effects, adsorption energetics, diffusion behavior, or coarse‐grained process observables. In this sense, ML does not displace computational chemistry; rather, it extends its reach by enabling surrogate prediction, descriptor compression, uncertainty‐aware screening, and prioritization of the most informative regions of chemical and process space for higher‐level study.

Hybridization is most defensible when the physical role of ML is explicit. DFT, MD, COSMO‐RS, or kinetic outputs can serve as chemically informed descriptors; ML can emulate an expensive particle or molecular sub‐model; and active learning can select the next high‐value experiment or simulation. These uses constrain the statistical model with a physically defined target and can reduce the amount of behavior learned solely from correlation.

### 7.4 Process Optimization, Digitalization, and Current Limitations

At the process level, ML is increasingly used not only for offline optimization but also for soft sensing, real‐time monitoring, hybrid modeling, and digital‐twin‐enabled operation. Parveen and Slater emphasized that catalyst optimization for biomass‐derived molecules cannot be separated from data‐rich experimentation, flow systems, and real‐time analytics [61]. In parallel, Butean et al. highlighted the broader role of AI in biorefineries and bioprocessing, particularly in soft sensing, hybrid modeling, digital twins, and real‐time control [134]. Coronado‐Contreras et al. extended this logic to digitally connected and sustainable biorefineries, positioning AI and related digital tools as enabling technologies for value‐chain integration and adaptive decision‐making [5]. This process‐side role is illustrated in Figure 10c, where ML serves as a soft‐sensing and prediction layer linking operational variables, monitored signals, and performance indicators in near real time. Although the figure uses anaerobic digestion as a representative example, the wider implication is general: ML is increasingly valuable not only because it predicts yields or compositions, but because it can function as an operational layer linking sensors, experiments, and process models in digitally connected biomass‐conversion systems.

Yet persistent limitations remain. Huang et al. and Jiang et al. both stressed that data scarcity, poor standardization, and weak interoperability continue to restrict robust ML deployment across lignocellulosic biorefineries [39, 54]. The problem is especially evident in pyrolysis, where Pascarella et al. showed that prediction quality and explainable‐AI interpretation depend strongly on missing‐data handling and dataset curation [95], while Yang et al. argued that predictive accuracy in pyrolysis has advanced faster than mechanistic trustworthiness [65]. Future progress will therefore depend less on ever‐larger black‐box models than on better datasets, uncertainty‐aware workflows, and hybrid models that embed chemical and physical knowledge.

### 7.5 Toward Autonomous and Integrated Workflows

The most compelling long‐term role of ML in biomass valorization lies in developing autonomous, integrated workflows that connect data generation, model updating, experiment selection, and process control within closed design loops. However, a critical assessment of current progress reveals a significant gap between aspiration and reality. A practical maturity hierarchy distinguishes this progression: Level I, retrospective cross‐validation; Level II, external validation on an independent feedstock, solvent, catalyst, or study; Level III, prospective experimental validation of ML‐selected candidates; and Level IV, sustained process‐integrated deployment. Currently, the majority of biomass ML research remains anchored in Levels I‐II, with only a few studies reaching Level III, and remarkably few biomass‐specific examples demonstrating sustained Level IV operation in real environments.

This stagnation at lower maturity levels signals that progress should be evaluated less by incremental increases in retrospective R^2^ and more by transferability, uncertainty awareness, physical consistency, and prospective validation. Crucially, negative or unsuccessful predictions are particularly valuable because they define failure boundaries and expose missing descriptors. In this role, ML does not replace mechanistic modeling or experiment; rather, it accelerates expensive calculations, integrates heterogeneous descriptors, prioritizes measurements, and constructs tractable surrogates for higher‐scale models. Yet achieving this aspirational Level IV autonomous, closed‐loop operation requires more than algorithmic refinements; it demands a fundamental overhaul of how we architect our research pipelines.

Accordingly, the next step is not simply more ML, but better‐connected ML. As articulated across the literature, this transition hinges on structured data frameworks and interoperable digital infrastructures. Butean et al. described a movement toward semi‐autonomous or autonomous biorefinery operation through hybrid modeling, advanced control, and AI‐enabled monitoring [134]. Coronado‐Contreras et al. similarly framed digitalization as an end‐to‐end systems architecture linking biomass resources, conversion routes, sustainability metrics, and operational decisions [5]. At the catalysis level, Parveen and Slater argued that such interoperable infrastructures are prerequisites for meaningful AI‐assisted discovery [61], while Moshantaf et al. demonstrated that FAIR‐compliant local data infrastructures can already support automated, machine‐readable catalysis workflows at laboratory scale [135]. Ultimately, ML becomes most valuable when it functions as an integrative layer linking experiments, simulations, and decision‐making requiring models built on curated datasets, physically meaningful descriptors, interpretable architectures, and digital infrastructures fully compatible with real process development and scale‐up.

## 8 Multiscale Integration Frameworks for Biomass Valorization

**FIGURE 11:**
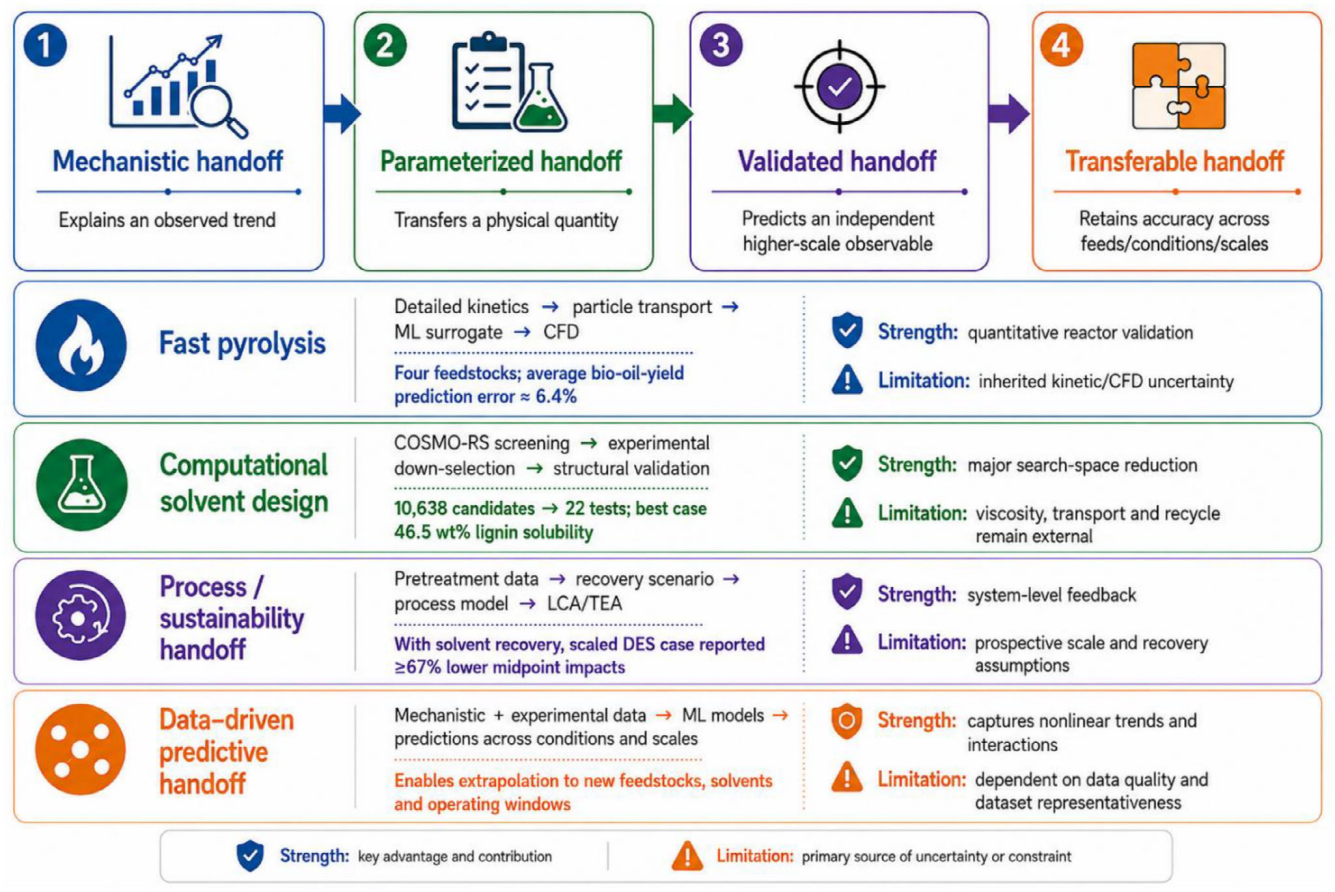
Multiscale integration maturity and representative quantitative handoffs in lignocellulosic biomass valorization. The framework progresses from mechanistic and validated handoffs to transferable prediction, with increasing integration stringency. Representative case studies anchor this progression: (i) coupled kinetics, particle transport, ML surrogates, and CFD in fast pyrolysis; (ii) computational solvent screening with experimental down‐selection; (iii) sustainability feedback via recovery‐aware TEA/LCA; and (iv) transferable prediction across feedstocks, solvents, and scales. For each case, the principal strength and limitation are highlighted, underscoring that success depends on validation, uncertainty control, and transferability.

Biomass valorization becomes predictive only when molecular, mesoscale, reactor, and system‐level descriptions are coupled through physically meaningful information handoffs rather than merely placed in the same workflow [6, 45]. Figure 11 summarizes the distinction introduced here between mechanistic, parameterized, validated, and transferable integration. A multiscale workflow should specify what quantity crosses each boundary, how that quantity is validated at the receiving scale, and what uncertainty or domain of validity information accompanies it.

The overall architecture remains iterative rather than strictly bottom‐up. Molecular and kinetic models provide descriptors for reactors and process systems, while higher‐scale sensitivity, TEA, and LCA should feedback to determine which lower‐scale quantities are worth refining. For example, if solvent recovery dominates the environmental burden, recovery behavior should be included in the solvent‐screening objective; if uncertainty in a particular kinetic parameter dominates bio‐oil yield, molecular calculations or experiments should target that parameter.

### 8.1 Hierarchical Coupling Across Scales

Multiscale modeling in biomass valorization is commonly organized hierarchically, with each level providing inputs to the next. At the smallest scale, electronic structure methods such as DFT resolve bond activation, adsorption energetics, and transition states. These outputs inform mesoscale approaches, including classical and reactive MD, which capture solvation, structural organization, diffusion, and evolving reactive environments. Larger‐scale kinetic and reactor models then translate these descriptors into observable rates, selectivity trends, transport limitations, and reactor performance, while TEA and LCA determine whether the resulting process is economically and environmentally defensible [6, 45]. The central challenge, however, is not merely to stack methods in sequence but to ensure that information transferred across scales remains physically meaningful. Activation barriers from DFT are useful in reactor models only when embedded within realistic kinetic schemes, and mesoscale structural data improve continuum descriptions only when they preserve the transport, accessibility, or phase behavior features that actually control conversion. Recent multiscale studies therefore frame scale coupling increasingly as a problem of representation fidelity rather than computational completeness [6, 45]. An integrated framework is valuable only when it preserves the structural or mechanistic feature that governs the engineering observable of interest.

Given this landscape, assessing whether a given multiscale implementation achieves meaningful coupling requires more than simply listing the methods used. The strength of cross‐scale handoffs can be classified along a maturity hierarchy. A *mechanistic handoff* provides a lower‐scale explanation for an observed trend without quantitatively parameterizing the next model. A *parameterized handoff* transfers a numerical quantity such as an activation barrier, diffusivity, activity coefficient, kinetic parameter, or particle response. A *validated handoff* occurs when the coupled model predicts an independent observable at the receiving scale and is compared with experiment. A *transferable handoff* requires that predictive performance remains acceptable across feedstocks, conditions, catalyst/solvent families, or equipment scales not used for development. This hierarchy prevents aspirational integration from being confused with demonstrated prediction. Validated adjacent‐scale coupling is increasingly feasible in biomass research, but robust atom‐to‐industrial transferability remains uncommon. The practical objective is therefore not to include the maximum number of computational techniques, but to connect the minimum set of representations required by the target engineering observable.

### 8.2 Integration of Computational and Data‐Driven Methods

Machine learning has emerged as a major enabler of multiscale integration because it can act as a surrogate, translator, or data‐fusion layer between otherwise disconnected models. Huang et al. reviewed ML across the lignocellulosic biorefinery pipeline and concluded that hybrid and transfer learning strategies are particularly important, as they help alleviate data scarcity while improving interpretability and extrapolation [39]. From a broader biomass‐valorization perspective, Jiang et al. identified three principal ML roles: basic data generation, process modeling, and multi‐objective system optimization [54]. These studies show that ML is most useful not when it replaces physics‐based modeling, but when it accelerates and connects it. This role is especially important in biomass valorization because the relevant information is highly heterogeneous. Experimental measurements, DFT‐derived descriptors, MD outputs, reactor data, TEA metrics, and environmental indicators are rarely generated in compatible formats. Yet, they must often be interpreted together to guide process design. From the perspective of catalytic production of bio‐based chemicals, Parveen and Slater argued that digital approaches are increasingly required to explore wider chemical space and accelerate sustainable discovery [61]. Coronado‐Contreras et al. extended this logic to smart and sustainable biorefineries by highlighting AI, IoT, and related digital tools as enabling technologies for integrating large, heterogeneous data streams across the biomass value chain [5]. In this sense, ML functions not only as a predictive tool, but also as an interoperability layer linking fragmented sources of chemical and engineering information.

Operationally, this interoperability operates through well‐defined quantitative handoffs across scales. Electronic‐structure calculations can provide activation free energies, adsorption energies, reaction energies, and electronic descriptors to kinetic or ML models. MD can provide diffusion coefficients, accessible area, aggregation measures, solvent configurations, or interfacial descriptors to transport, solvent, or ML models. Reactive simulation can provide candidate reaction motifs for higher‐level energetic refinement and network reduction. Thermodynamic screening can provide relative affinity and phase‐behavior information for experimental down‐selection and separation analysis. Kinetic schemes provide rate expressions to particle and reactor models, while reactor models provide conversion, selectivity, heat duty, residence‐time requirements, and product composition to process simulation. ML can operate within these interfaces as a surrogate, translator, prioritization layer, or model‐discovery tool. Its most defensible scale‐bridging role occurs when the target is physically defined, as in a surrogate trained on particle simulations for use in CFD [60], rather than when a black‐box output is passed directly to a downstream model without domain or uncertainty information.

### 8.3 Coupling With Process and Reactor Models

For multiscale frameworks to move beyond conceptual diagrams, they must connect directly to process and reactor models that reflect realistic operating conditions. This requirement is particularly clear in pyrolysis, where Naidu et al. showed that the most useful recent models combine reaction kinetics with CFD, heat transfer, and reactor‐scale transport to improve predictions of scale‐up and energy efficiency [45]. Their analysis underscores that chemistry‐only models become insufficient once particle‐scale transport, residence‐time distribution, and reactor configuration begin to shape the final product slate. The same principle applies to catalytic conversion and fractionation systems. A framework that links active‐site chemistry to catalyst formulation but neglects diffusion limitations, deactivation, solvent effects, or phase behavior remains only partially predictive. Martín et al. similarly argued that biomass composition and resource distribution must be connected directly to process design [6]. At the same time, recent studies on digitalization further show that reactor and process models increasingly interact with real‐time data, soft sensors, and surrogate models [61, 136]. Multiscale integration, therefore, becomes meaningful only when molecular chemistry is translated into decision‐relevant process behavior.

Three case studies illustrate different degrees of this integration in practice. First, Lu et al. coupled detailed fast‐pyrolysis kinetics with an intraparticle model, trained an ML surrogate for particle behavior, and embedded that surrogate in a fluidized‐bed CFD model; the resulting workflow yielded an average bio‐oil yield prediction error of approximately 6.4% across four feedstocks [60]. This represents a validated handoff with partial evidence of transferability, although uncertainties in kinetic, particle, surrogate, and CFD remain. Second, Pontes et al. used COSMO‐RS to screen 10 638 eutectic‐solvent combinations, reduced the space to 22 experimentally tested systems, and identified a best‐performing case with 46.5 wt% lignin solubility and 111% relative laccase activity [51]. The principal handoff is molecular thermodynamic ranking to experimental candidate selection. This is a strong validated adjacent‐scale workflow, but viscosity, transport, biomass morphology, water content, and recycle remain outside the screening model. Third, the prospective DES analysis discussed in Chapter 4 transferred laboratory pretreatment and solvent‐use information into process‐scale recovery scenarios and LCA. Incorporation of solvent recovery and scale‐up produced at least 67% lower reported midpoint impacts relative to the laboratory‐scale configuration [13]. This case shows why process/system analysis should feed back into molecular design, while also illustrating the sensitivity of prospective conclusions to assumptions about recovery, energy, and scale (Table 4).

**TABLE 4:** Representative multiscale integration pathways in predictive biomass valorization.

Case	Scale sequence	Information transferred	Quantitative outcome	Integration maturity	Main limitation
Fast pyrolysis [60]	Kinetics → particle → ML surrogate → CFD → experiment	Reaction rates and particle‐scale responses	∼6.4% average bio‐oil yield error across four feedstocks	Validated; partial transferability	Inherited uncertainty from kinetic, particle, and CFD models
Eutectic solvent screening [51]	Molecular thermodynamics → ranking → experiment	Relative solvent affinity	10 638 candidates → 22 tests; 46.5 wt% lignin solubility; 111% relative laccase activity	Validated at adjacent scales	Transport effects, real‐biomass structure, and recycle omitted
DES structural design [49]	Computational solvent descriptors → pretreatment → lignin analysis	Solvent chemistry → linkage response	Differential β‐O‐4 preservation / cleavage	Mechanistically validated	Limited transferability to other feedstocks or processes
DES process / LCA [13]	Lab pretreatment → process/recovery → LCA	Yield, solvent demand, recovery	≥67% lower reported midpoint impacts after scale/recovery integration	Parameterized system handoff	Prospective assumptions inherent in LCA
FAIR catalytic workflow [135]	Experiment → metadata capture → database / API	Data, metadata, provenance	Machine‐readable automated workflow demonstrated	Digitally integrated	Currently laboratory‐scale; not yet extended to full biorefinery

### 8.4 Digitalization, FAIR Data, and Workflow Automation

The development of integrated multiscale frameworks is now closely tied to digitalization and workflow automation. Gamero et al. introduced the concept of a Lean Digital Twin for biorefineries and argued that a digital twin informed by continuous process data can improve efficiency and reduce adverse impacts without imposing unrealistic requirements for exhaustive sensing and data acquisition [136]. This is especially important because it reframes digital twins not as abstract future tools, but as operational platforms designed around realistic data availability. A second crucial development is the growing emphasis on data infrastructure. Moshantaf et al. demonstrated in an automated catalytic test reactor that FAIR data practices can be implemented through automated data acquisition and metadata, machine‐readable standard operating procedures, and APIs linking local databases to broader repositories; they identified this as a foundation for machine learning and autonomous catalyst discovery [135]. In parallel, Parveen and Slater showed that digitalized catalytic workflows are central to the sustainable production of bio‐based chemicals [61], while Butean et al. highlighted digital twins, soft sensing, and hybrid models as key components of real‐time control and optimization in biorefineries [134]. Workflow automation in biomass valorization, therefore, extends well beyond high‐throughput screening and increasingly depends on machine‐readable experimentation, structured metadata, and automated links among experiments, simulations, and optimization tools.

Despite this progress, several barriers still limit the development of robust multiscale frameworks. One of the most persistent is uncertainty propagation across scales. Small errors in molecular energetics, force‐field parameterization, or inferred kinetic parameters can be amplified when passed into reactor, TEA, or LCA models. Martín et al. and Naidu et al. both make clear that successful integrated biomass models require more than coupling; they require consistent transfer of assumptions, validity limits, and uncertainty across levels of description [6, 45]. A second major barrier is data standardization. Huang et al. showed that hybrid ML strategies are attractive partly because they compensate for sparse and fragmented datasets. Still, they also underscored the continued need for robust model development and evaluation across the lignocellulosic biorefinery pipeline [39]. Moshantaf et al. went further by showing that FAIR data are becoming indispensable as AI workflows demand more reliable, reproducible, and machine‐actionable datasets [135]. Coronado‐Contreras et al. likewise emphasized that automated and digitally integrated biomass systems require validation, uncertainty quantification, and compatibility across multiple tools [5]. The principal bottleneck is therefore no longer the absence of methods but the lack of interoperable, uncertainty‐aware workflows that link them.

A practical strategy to address this bottleneck is sensitivity‐driven refinement. Higher‐scale reactor, TEA, or LCA models can identify which lower‐scale variables control the final decision. High‐fidelity calculations and experiments can then be concentrated on the dominant uncertain descriptors. This top‐down feedback is more efficient than uniformly increasing model fidelity scale across all scales and makes decision‐oriented accuracy a more useful standard than maximal resolution. Uncertainty should therefore be treated as part of the scale handoff rather than accumulated silently; passing only a best‐estimate value can create an appearance of increasing precision even as uncertainty grows.

### 8.5 Toward Predictive and Design‐Oriented Frameworks

The long‐term goal of multiscale integration in biomass valorization is not simply more detailed simulation, but genuinely predictive and design‐oriented biorefinery development. Huang et al. argued that ML should be deployed across the entire lignocellulosic workflow rather than within isolated sub‐processes [39], while Butean et al. described a broader shift toward AI‐supported optimization, real‐time monitoring, and increasingly autonomous or semi‐autonomous decision‐making in biorefineries [134]. Coronado‐Contreras et al. extended this perspective to smart and sustainable biorefineries, emphasizing AI, IoT, and digital tools as part of a more integrated circular‐bioeconomy framework [5]. These studies indicate that future frameworks will be hybrid by default: mechanistic, where chemistry and transport matter most; data‐driven, where speed and adaptability are essential; and system‐level, where economics and sustainability ultimately determine viability. The recent literature, therefore, supports a clear conclusion that multiscale integration is becoming a key measure of maturity in biomass valorization modeling. Martín et al. frame this as a systems requirement for lignocellulosic biorefineries [6], Naidu et al. show its necessity in pyrolysis and reactor design [45], and Huang et al., Parveen and Slater, Butean et al., Gamero et al., and Moshantaf et al. collectively show that the field is moving toward hybrid, digitalized, and increasingly automated workflows [39, 61, 134, 135, 136].

The remaining challenge is to ensure that these workflows are not only connected but also interpretable, uncertainty‐aware, and grounded in machine‐readable data practices. The overarching objective is therefore a modular, interoperable, and uncertainty‐aware modeling ecosystem rather than one monolithic simulation spanning electrons to commercial plants. Each module should have defined inputs and outputs, an experimental validation target, a versioned provenance record, and an explicit domain of applicability. Progress should ultimately be measured by how successfully knowledge survives transitions between scales, not by how many methods appear in a workflow.

## 9 Data Infrastructure, FAIR Principles, and Digitalization

**FIGURE 12:**
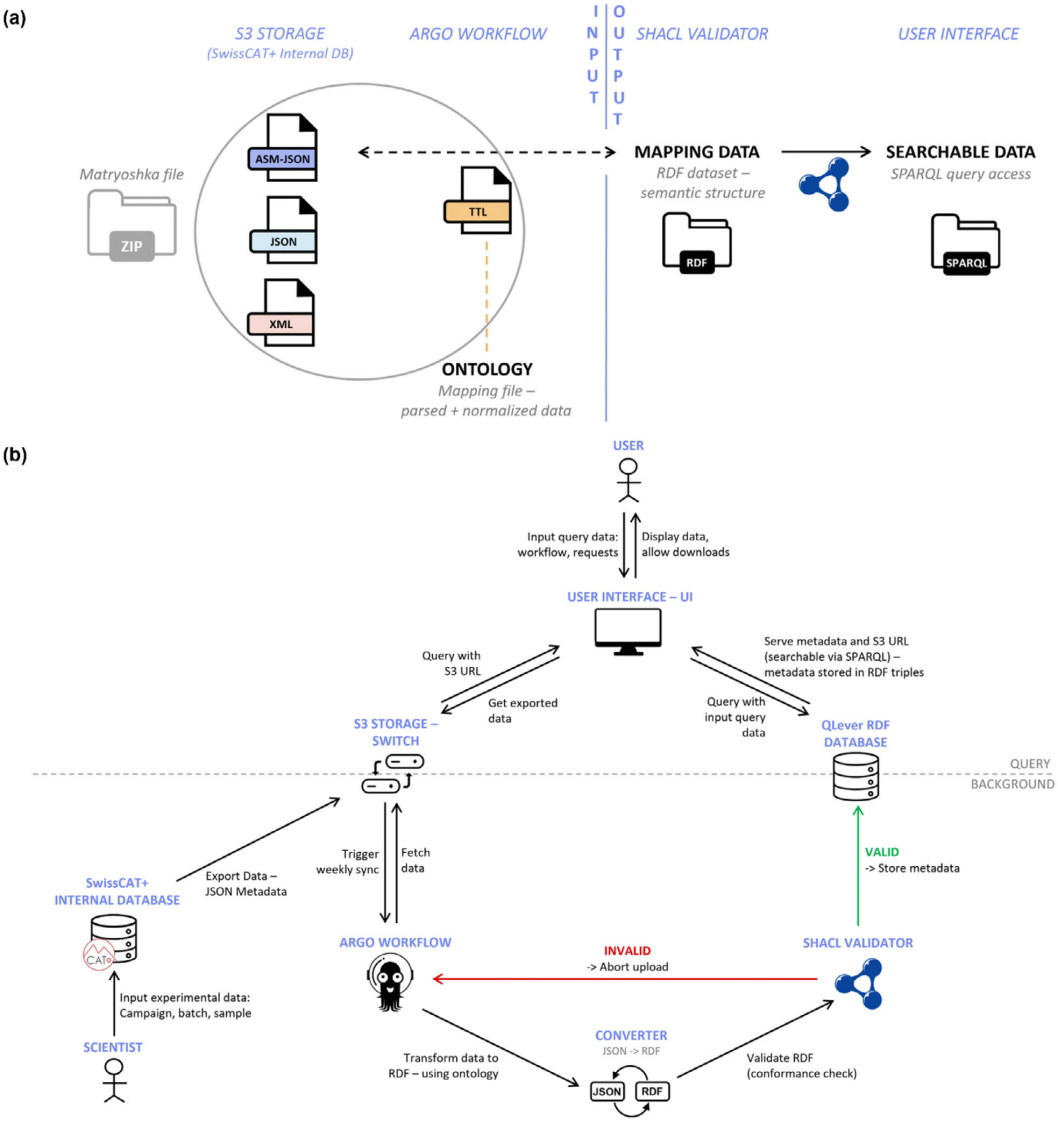
(a) Swiss Cat+ research‐data infrastructure for transforming heterogeneous and nested experimental files into semantically structured and queryable RDF graphs; (b) prospective development toward AI‐ready data and interoperability with external laboratory infrastructures. Panels (a,b) reproduced from Ref. [69] under the terms of the Creative Commons Attribution 3.0 Unported Licence (CC BY 3.0). 2025 The Authors. Published by the Royal Society of Chemistry.

Data infrastructure is emerging as a decisive scientific enabler in biomass valorization because predictive workflows now depend on the capture, standardization, and reuse of heterogeneous experimental, computational, and process data [5, 61]. As shown in Figure 12, this requires more than data storage: it demands interoperable architectures that link structured packaging, workflow orchestration, machine‐readable experimental management, archive governance, and digital‐physical traceability into a single ecosystem. In this framework, FAIR data practices are not an administrative ideal, but the foundation for reproducible modeling, automated experimentation, and cross‐scale decision‐making.

The scope of this chapter is distinct from Chapter 7: Chapter 7 addresses how ML models learn from data, whereas Chapter 9 addresses how reliable data are identified, structured, exchanged, and maintained so that those models and multiscale workflows can be reproduced. A useful digital architecture realizing these goals follows a structured pipeline: physical sample or simulation → raw data object → contextual metadata → standardized vocabulary/ontology → validated machine‐readable representation → searchable repository or knowledge graph → API/workflow exchange → downstream model or process application, with provenance and uncertainty retained throughout. FAIR data therefore means more than placing a spreadsheet in an online repository. The central implication is that digitalization in biomass valorization will be determined less by the availability of algorithms than by the ability to build traceable, machine‐actionable, and uncertainty‐aware data systems that connect methods, metadata, samples, and process histories across the biorefinery value chain.

### 9.1 FAIR Data as a Prerequisite for Predictive Biomass Research

The FAIR (Findable, Accessible, Interoperable, and Reusable) principles have become the dominant framework for organizing scientific data so that they remain useful beyond the project in which they were first generated. In catalysis and chemistry, recent studies emphasize that FAIR implementation requires more than data sharing alone; it also requires machine‐readable data, metadata, and workflows that preserve context, provenance, and reusability [135, 137]. This point is especially important in biomass valorization, where predictive models often combine experimental measurements, simulation outputs, and process‐level indicators generated under very different conditions. Under these circumstances, FAIR compliance is not simply a matter of good record‐keeping but a prerequisite for building trustworthy datasets that can support validation, benchmarking, transfer learning, and cross‐platform model development.

Operationally, the FAIR principles provide a practical framework for evaluating data architecture. *Findability* requires persistent identity and structured metadata; *accessibility* requires documented retrieval and access rules; *interoperability* requires common formats, schemas, identifiers, and semantic meaning; and *reusability* requires enough contextual information to reconstruct what was measured or calculated, on which sample, by which method, under which conditions, and with what uncertainty. Figure 12a,b provides a concrete interpretation of these principles, showing that findability and accessibility depend on structured packaging and searchable interfaces. In contrast, interoperability and reusability depend on metadata normalization, semantic conversion, validation, and workflow‐level consistency.

For biomass research specifically, these requirements extend well beyond conventional reaction records. Feedstock provenance, tissue fraction, storage and drying history, particle size, moisture, ash, composition, pretreatment history, solvent water content, catalyst synthesis and aging, reactor configuration, analytical method, units, uncertainty, and relationships among derived fractions are often necessary to interpret a numerical response. FAIR data therefore requires more than repositories; it requires infrastructures that preserve the relationships among experiments, methods, samples, and outputs in machine‐readable form.

### 9.2 Data Generation, Curation, and Metadata Quality

**FIGURE 13:**
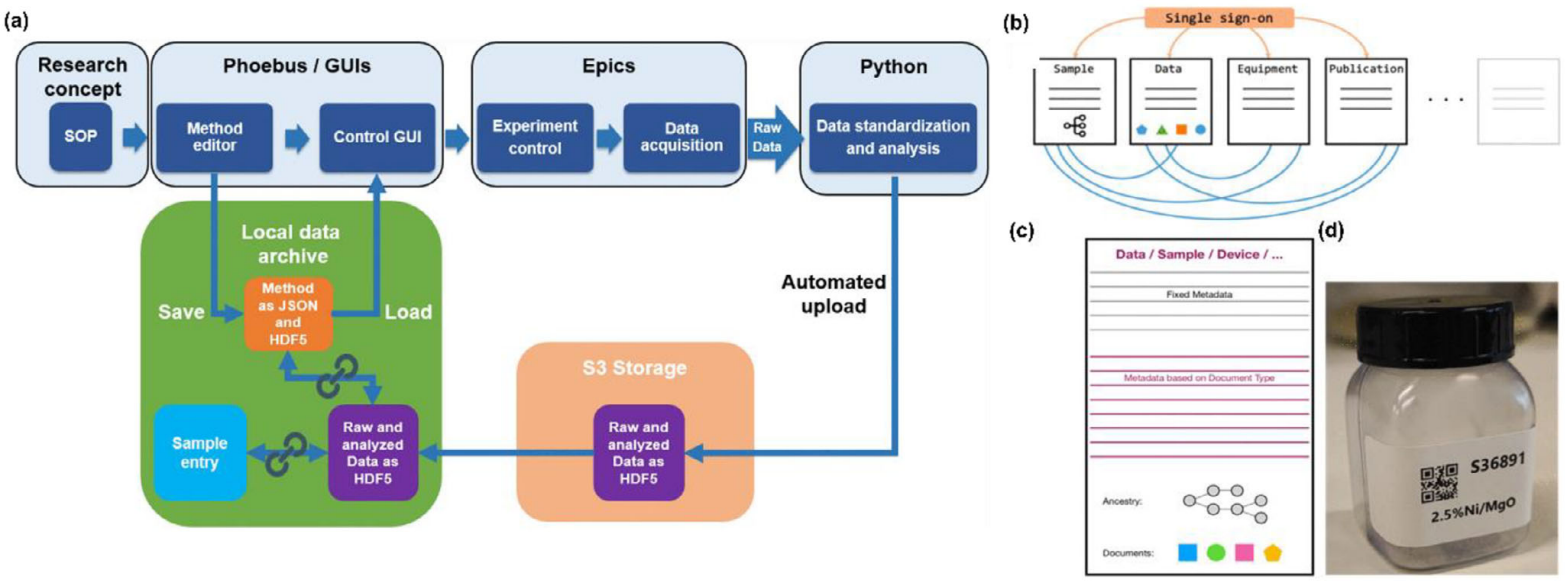
(a) Machine‐readable standard operating procedures and associated FAIR data‐governance workflow for automated catalysis research. Reproduced from Ref. [135] under the terms of the Creative Commons Attribution 3.0 Unported Licence (CC BY 3.0). 2024 The Authors. (b) Adaptable AC/CATLAB Archive architecture supporting diverse research‐document types; (c) minimal‐field document design for standardized data capture; (d) QR‐coded physical‐sample labels enabling direct access to associated digital records. Panels (b–d) reproduced from Ref. [68] under the terms of the Creative Commons Attribution 3.0 Unported Licence (CC BY 3.0). 2023 The Authors.

Data in biomass valorization originate from diverse and often incompatible sources, including wet lab experiments, spectroscopy and chromatography, DFT calculations, molecular simulations, kinetic models, and process simulations. The central challenge is therefore not only data volume, but also curation and contextualization. Moshantaf et al. showed that automated acquisition of both data and metadata can be implemented through machine‐readable standard operating procedures, automated database uploads, and API‐mediated exchange across repositories [135]. Complementarily, Husková et al. proposed a use‐case‐driven methodology for improving data and metadata quality in catalysis, highlighting the importance of ontology and vocabulary mapping, semantic representation, and collaboration with domain researchers to make datasets machine‐readable and cross‐linkable [138]. For biomass valorization, effective curation must therefore preserve not only numerical values, but also the experimental conditions, assumptions, and provenance that make those values scientifically reusable. This logic is visualized directly in Figure 13a, where method files, raw measurements, analyzed outputs, and archive entries are linked within a common workflow. The significance of this arrangement is that it converts isolated measurements into connected experimental histories. In biomass valorization, where predictive reliability depends strongly on process context, such linkage is not a convenience but a precondition for meaningful reuse and comparison.

Negative or unsuccessful outcomes should also be retained. Solvents that fail to fractionate biomass, catalysts that deactivate rapidly, unstable solvent formulations, failed phase separations, and prospective ML candidates that fail to validate experimentally help define the boundaries of applicability and reduce publication bias. Data quality governance should therefore combine machine‐actionable schemas with unit checks, controlled vocabularies, range/plausibility rules, calibration records, and human‐auditable exceptions.

### 9.3 Repositories, Ontologies, and Linked Knowledge Systems

The development of centralized or interoperable knowledge repositories is increasingly important for data‐driven biomass research. Recent work on digital chemistry infrastructure argues that chemistry still lags behind some data‐intensive fields because datasets remain incomplete, weakly standardized, and often biased toward successful experiments, with detailed negative data still rarely captured [69]. In response, FAIR‐oriented research data infrastructures are being designed to transform fragmented research outputs into reusable, semantically structured resources. Related efforts, such as the Open Reaction Database, provide open, machine‐readable reaction data for model development [139], while semantic wiki‐based chemistry knowledge bases have demonstrated that structured formats and machine‐readable chemical representations can support the collaborative collection and comparison of research results [140]. At a higher semantic level, recent reviews of knowledge graphs in heterogeneous catalysis conclude that these frameworks can integrate dispersed information on materials, synthesis, mechanisms, and reaction conditions in line with FAIR principles, but also note ongoing challenges in ontology alignment, heterogeneous data integration, and long‐term maintenance [66, 141]. For biomass valorization, the implication is clear: future repositories will need to function not as static archives, but as linked knowledge systems that support search, inference, and model training. Figure 13b illustrates the transition from heterogeneous source files to validated, searchable, semantically structured data objects.

Several concrete resources illustrate the required components even though they are not yet a complete biomass‐specific ecosystem. Swiss Cat+ demonstrates transformation of heterogeneous files through normalization, ontology mapping, RDF representation, semantic validation, and queryable storage [69]. The Open Reaction Database provides machine‐readable reaction records [139]. Ontologies4Cat and related vocabulary‐mapping efforts address formal definition and alignment of catalysis concepts [67, 138], while semantic wikis and knowledge graphs provide routes for linking heterogeneous entities [140, 141]. These platforms should be interpreted as enabling precedents rather than as evidence that an end‐to‐end FAIR lignocellulosic biorefineries database already exists.

### 9.4 Digital Workflows, Automation, and Interoperability

Digitalization in biomass valorization increasingly involves more than digitizing individual experiments; it involves building automated workflows that connect experiments, simulations, databases, and optimization tools into iterative learning loops. Moshantaf et al. provided a concrete example by demonstrating that automated catalytic‐test infrastructure can capture data and metadata, standardize analysis, and exchange information via APIs, thereby laying the foundations for ML‐enabled, potentially autonomous discovery workflows [135]. In the broader context of catalytic biomass conversion, Parveen and Slater argued that digitalized approaches are essential for exploring wider chemical space and for connecting catalyst design, synthesis, characterization, and process optimization [61]. Related reviews on biomass digitalization and biorefinery operations likewise identify AI, Big Data, digital twins, soft sensing, and hybrid models as foundational technologies for predictive analytics, automation, and improved decision‐making [5, 66]. However, interoperability remains a persistent barrier. Both Husková et al. and the Ontologies4Cat study emphasize that data and metadata must be standardized and semantically represented to be reused across workflows, while recent knowledge graph analyses underscore the ongoing challenges of ontology discovery, reuse, and long‐term graph maintenance in complex catalysis domains [67, 138, 141]. In biomass valorization, where catalyst data, solvent properties, reaction networks, TEA outputs, and environmental indicators often need to be connected, common schemas, ontology‐aware metadata, and open APIs provide the practical infrastructure that enables cross‐scale integration. This workflow logic is visualized in Figure 13, where semantic conversion, synchronization, machine‐readable acquisition, and archive linkage appear as connected layers rather than isolated data steps. Figure 13b–d extends the same logic into governance and laboratory practice by showing that digital workflows require not only technical interoperability but also traceable sample identity, controlled access, and persistent links between physical materials and digital records. As digital and ML‐enabled workflows become more prominent, uncertainty quantification is also becoming part of data infrastructure rather than a downstream analytical refinement. In chemistry ML, Heid et al. showed that prediction error can arise from distinct sources, including data noise, model bias, and model variance, and that these sources must be distinguished to improve reliability [66]. At the interface of ML and sustainability analysis, Akrami et al. similarly argued that uncertainty must be explicitly propagated and managed when ML is integrated with life‐cycle assessment [142]. This issue is especially important in biomass valorization because uncertainty can originate at every stage, including feedstock variability, experimental reproducibility, simulation assumptions, missing metadata, and model extrapolation. A mature data infrastructure must therefore preserve uncertainty information, provenance, and confidence limits alongside values and metadata so that downstream decisions remain scientifically defensible.

Interoperability can be separated into four practical levels. File interoperability means that another system can open the data; syntactic interoperability means that records follow a predictable schema; semantic interoperability means that variables such as yield, conversion, lignin type, or pretreatment severity have formally defined meanings; and workflow interoperability means that data can move among instrument, database, analysis software, simulation, ML, and process models without manual reinterpretation. The Swiss Cat+ RDF architecture in Figure 12 illustrates the transition from files to semantic interoperability, while the automated reactor infrastructure discussed below illustrates workflow‐level exchange.

Moshantaf et al. demonstrated FAIR principles in an automated catalytic test reactor by connecting machine‐readable SOPs, experimental control, automated metadata capture, database upload, and API‐mediated exchange [135]. The AC/CATLAB‐style architecture shown in Figure 13 adds digital‐physical traceability through persistent sample records and QR‐linked material identity [68]. This is particularly relevant to biomass, where one feedstock may generate a lineage of pretreated solids, isolated lignin, hydrolysates, catalyst‐tested fractions, and residues that must remain linked digitally. Table 5 summarizes the practical FAIR‐data requirements that support reproducible and interoperable biomass workflows, together with representative enabling resources and the corresponding biomass‐specific needs.

**TABLE 5:** Practical FAIR data requirements for predictive lignocellulosic biomass valorization.

Requirement	Practical implementation	Representative resource	Biomass‐specific need
Findability	Persistent identifiers; indexed metadata; searchable fields	Swiss Cat+ [69]	Feedstock provenance and pretreatment metadata must be searchable
Accessibility	Repository / API with explicit access rules	Open Reaction Database [139]	Programmatic access to biomass‐conversion datasets
Syntactic interoperability	Standard JSON / XML / RDF schemas	Swiss Cat+ [69]	Common schemas spanning pretreatment, catalysis, and reactor operations
Semantic interoperability	Ontologies; controlled vocabularies; RDF relationships	Ontologies4Cat [67]; Swiss Cat+ [69]; metadata mapping [138]	Biomass‐specific terminology for feedstock, fractionation, and lignin structure
Reusability	Retain methods, units, conditions, uncertainty, and provenance	FAIR automated reactor workflow [135]; data‐resource perspective [137]	Link data to analytical and process histories
Physical traceability	Digital sample records with persistent sample IDs	AC / CATLAB architecture [68]	Track feedstock‐derived fractions across processing stages
Automation	Machine‐readable SOPs; automated upload; APIs	Mosshantaf et al. [135]	Integrated pretreatment, catalysis, and analysis workflows
Linked knowledge	Semantic wiki/knowledge graph	Semantic Wiki [140]; knowledge graphs [141]	Connect structure → processing → product → process metrics
Uncertainty‐aware data	Store confidence/quality metadata alongside values	Uncertainty methods [66, 142]	Propagate uncertainty across molecular‐to‐process models
Digital operation	Model/process synchronization	AI‐enabled process operation [134]; Lean Digital Twin [136]	Validated process twins with clearly defined model domains

### 9.5 Toward Fully Digitalized Biorefineries

The convergence of FAIR data practices, interoperable repositories, automated workflows, and uncertainty‐aware analytics is gradually creating the basis for increasingly digitalized biorefineries. Coronado‐Contreras et al. framed digital twins, AI, and Big Data as key tools for improving circularity and efficiency in biomass systems [5], while FAIR‐oriented infrastructures in catalysis and digital chemistry further reinforce this trajectory [69, 135]. Gamero et al. introduced the concept of a Lean Digital Twin for biorefinery optimization, arguing that useful process representations can be derived from available operational data, even when exhaustive instrumentation is impractical [136]. Such process‐level twins may combine sensors, mass and energy balances, kinetic or surrogate models, equipment states, soft sensors, and optimization routines.

Claims regarding technological maturity should nevertheless be carefully calibrated. Process‐level digital twins are becoming increasingly feasible, whereas continuously updating molecular to process digital twins that integrate biomass structure, DFT/MD simulations, catalyst chemistry, reactor‐scale CFD, TEA/LCA, and plant sensor data remain largely aspirational for lignocellulosic systems. Existing FAIR data resources, automated laboratory workflows, semantic knowledge representations, and process‐level digital twins provide important enabling components. However, the principal infrastructure challenge remains interoperability across the complete information lineage: feedstock → structure → treatment → fraction → conversion → reactor → process → sustainability outcome. The literature therefore supports a clear conclusion: robust data infrastructure is no longer a supporting element of biomass valorization research, but one of its principals enabling technologies. The remaining challenge is not whether digitalization will matter, but whether the field can build sufficiently standardized, machine‐readable, and uncertainty‐aware data ecosystems to make predictive biomass valorization genuinely scalable.

## 10 Conclusions and Outlook

Lignocellulosic biomass valorization is best understood as a multiscale design problem in which feedstock structure, molecular interactions, reaction chemistry, transport, reactor operation, separations, recycle, economics, and environmental performance interact to determine process outcome. Across this Review, DFT, molecular dynamics, reactive simulation, thermodynamic screening, kinetic modeling, machine learning, reactor simulation, TEA/LCA, and FAIR digital infrastructure are shown to be complementary rather than interchangeable. Their predictive value depends on whether the representation preserves the feature that controls the target observable, whether the prediction is validated against a corresponding experiment, and whether uncertainty and domain of applicability remain explicit as information is transferred to another scale.

Several adjacent‐scale interfaces are already demonstrated. Detailed pyrolysis kinetics have been coupled to particle‐scale transport, an ML surrogate, and CFD with an average bio‐oil‐yield prediction error of approximately 6.4% across four feedstocks [60]. COSMO‐RS‐assisted solvent design has reduced a space of 10 638 eutectic‐solvent combinations to 22 experimental candidates, with the best reported system reaching 46.5 wt% lignin solubility and 111% relative laccase activity [51]. At the process‐system interface, a prospective DES assessment showed that solvent recovery and scale‐up can materially alter sustainability conclusions, with reported midpoint impacts at least 67% lower than those of the laboratory‐scale configuration in the evaluated scenario [13]. These examples demonstrate that validated adjacent‐scale coupling is feasible, while complete and routinely transferable atom‐to‐industrial biorefinery workflows remain uncommon.

Five priorities follow from the evidence base. First, experimentally anchored structure‐property relationships should connect feedstock‐resolved characterization with controlled deconstruction and simulation, using crystallinity, lignin chemistry, porosity, and probe‐specific accessibility as conditional rather than universal descriptors. Second, computational‐experimental validation should be designed prospectively so that predicted solvents, catalysts, mechanisms, or process responses are tested independently, and unsuccessful predictions are retained as evidence of model boundaries. Third, solvents and catalysts should be evaluated over multiple recycle or regeneration cycles, including solvent compositional drift, catalyst time‐on‐stream, impurities, deactivation, separation burden, and carbon‐retention performance. Fourth, reactor, TEA, and LCA sensitivities should feed back into molecular design so that the variables that dominate cost, emissions, or yield determine where higher‐fidelity calculations and experiments are invested. Fifth, ML should be evaluated through external, grouped, and prospective validation with uncertainty and applicability‐domain information rather than retrospective R^2^ alone.

Progress also requires a FAIR biomass‐specific data ecosystem. The critical need is not necessarily one universal database, but interoperable resources that preserve feedstock provenance, processing history, analytical method, units, uncertainty, sample lineage, model version, and negative results through common schemas, ontologies, persistent identifiers, APIs, and provenance. Process digital twins can then become increasingly useful operational tools; fully adaptive molecular‐to‐process twins should be regarded as a longer‐term objective rather than a current routine capability.

Therefore, the defining objective for the next generation of lignocellulosic biorefinery research is the transition from retrospective explanation to experimentally validated, uncertainty‐aware prospective design. The most credible architecture is modular: mechanistic where chemistry matters, data‐driven where complexity and speed demand it, transport‐aware where scale alters performance, and system‐oriented where economics and sustainability determine viability. Computational and data‐driven strategies will become genuinely predictive when well‐defined knowledge can survive the transitions among these modules and alter real experimental and engineering decisions.

## Author Contributions

All authors have equally contributed to this review.

## Conflicts of Interest

The authors declare no conflicts of interests.

## Data Availability

No new primary research results have been included, and no new data were generated or analyzed as part of this review.
